# Parental education and inequalities in child mortality: a global systematic review and meta-analysis

**DOI:** 10.1016/S0140-6736(21)00534-1

**Published:** 2021-08-14

**Authors:** Mirza Balaj, Hunter Wade York, Kam Sripada, Elodie Besnier, Hanne Dahl Vonen, Aleksandr Aravkin, Joseph Friedman, Max Griswold, Magnus Rom Jensen, Talal Mohammad, Erin C Mullany, Solvor Solhaug, Reed Sorensen, Donata Stonkute, Andreas Tallaksen, Joanna Whisnant, Peng Zheng, Emmanuela Gakidou, Terje Andreas Eikemo

**Affiliations:** a Centre for Global Health Inequalities Research, Department of Sociology and Political Science, Norwegian University of Science and Technology, Trondheim, Norway; b Institute for Health Metrics and Evaluation, University of Washington, Seattle, WA, USA; c Department of Applied Mathematics, University of Washington, Seattle, WA, USA; d Department of Sociology and Office of Population Research, Princeton University, Princeton, NJ, USA; e Department of Health Metrics Sciences, School of Medicine, University of Washington, Seattle, WA, USA; f Center for Social Medicine and Humanities, University of California Los Angeles, Los Angeles, CA, USA; g RAND Corporation, Santa Monica, CA, USA; h Library Section for Humanities, Education and Social Sciences, University Library, Norwegian University of Science & Technology, Trondheim, Norway; i Department of Health Promotion, Norwegian Institute of Public Health, Bergen, Norway

## Abstract

**Background:**

The educational attainment of parents, particularly mothers, has been associated with lower levels of child mortality, yet there is no consensus on the magnitude of this relationship globally. We aimed to estimate the total reductions in under-5 mortality that are associated with increased maternal and paternal education, during distinct age intervals.

**Methods:**

This study is a comprehensive global systematic review and meta-analysis of all existing studies of the effects of parental education on neonatal, infant, and under-5 child mortality, combined with primary analyses of Demographic and Health Survey (DHS) data. The literature search of seven databases (CINAHL, Embase, MEDLINE, PsycINFO, PubMed, Scopus, and Web of Science) was done between Jan 23 and Feb 8, 2019, and updated on Jan 7, 2021, with no language or publication date restrictions. Teams of independent reviewers assessed each record for its inclusion of individual-level data on parental education and child mortality and excluded articles on the basis of study design and availability of relevant statistics. Full-text screening was done in 15 languages. Data extracted from these studies were combined with primary microdata from the DHS for meta-analyses relating maternal or paternal education with mortality at six age intervals: 0–27 days, 1–11 months, 1–4 years, 0–4 years, 0–11 months, and 1 month to 4 years. Novel mixed-effects meta-regression models were implemented to address heterogeneity in referent and exposure measures among the studies and to adjust for study-level covariates (wealth or income, partner's years of schooling, and sex of the child). This study was registered with PROSPERO (CRD42020141731).

**Findings:**

The systematic review returned 5339 unique records, yielding 186 included studies after exclusions. DHS data were compiled from 114 unique surveys, capturing 3 112 474 livebirths. Data extracted from the systematic review were synthesized together with primary DHS data, for meta-analysis on a total of 300 studies from 92 countries. Both increased maternal and paternal education showed a dose–response relationship linked to reduced under-5 mortality, with maternal education emerging as a stronger predictor. We observed a reduction in under-5 mortality of 31·0% (95% CI 29·0–32·6) for children born to mothers with 12 years of education (ie, completed secondary education) and 17·3% (15·0–18·8) for children born to fathers with 12 years of education, compared with those born to a parent with no education. We also showed that a single additional year of schooling was, on average, associated with a reduction in under-5 mortality of 3·04% (2·82–3·23) for maternal education and 1·57% (1·35–1·72) for paternal education. The association between higher parental education and lower child mortality was significant for both parents at all ages studied and was largest after the first month of life. The meta-analysis framework incorporated uncertainty associated with each individual effect size into the model fitting process, in an effort to decrease the risk of bias introduced by study design and quality.

**Interpretation:**

To our knowledge, this study is the first effort to systematically quantify the transgenerational importance of education for child survival at the global level. The results showed that lower maternal and paternal education are both risk factors for child mortality, even after controlling for other markers of family socioeconomic status. This study provides robust evidence for universal quality education as a mechanism to achieve the Sustainable Development Goal target 3.2 of reducing neonatal and child mortality.

**Funding:**

Research Council of Norway, Bill & Melinda Gates Foundation, and Rockefeller Foundation-Boston University Commission on Social Determinants, Data, and Decision Making (3-D Commission).

## Introduction

Education and child survival have both been at the heart of the international development agendas for decades,^1^, ^2^, ^3^ yet large inequalities in education attainment persist within and between countries,^4^ as do large inequalities in child mortality rates.^5^, ^6^ Despite decades of reports documenting education-related disparities in child survival, to our knowledge, no study to date has attempted to systematically quantify the effect of parental education on under-5 mortality on a global scale. Understanding how increased parental education might reduce child mortality rates and close within-country and between-country inequalities in child mortality is, therefore, of crucial importance to tackling social inequalities in health and achieving the Sustainable Development Goals (SDGs).

**Research in context**

**Evidence before this study**

We searched seven academic databases for studies that used individual-level data to estimate the relationship between inequalities in child mortality and parental education, with no restriction by language or date. Data from the 186 included articles were combined with data from 114 Demographic and Health Surveys (DHS) for meta-analysis.

In 2008, the Commission on the Social Determinants of Health underscored the link between education and improved health outcomes, both directly and through its effect on other determinants of health such as income, employment, and living conditions. It has remained challenging for researchers to disentangle the interacting effects of social factors, such as education, employment, urbanicity, wealth, and income, among others. Previous research has sought to quantify the effect of maternal education on child mortality, but reviewed only a portion of evidence available, namely from select DHS waves in low-income and middle-income countries. One 2010 study, drawing on data from 175 countries, estimated that half the reduction in child mortality since 1970 could be attributed to increased educational attainment among women, with use of country-level covariates for income per person and HIV seroprevalence. Another study, from 2011, found that increased maternal education improved the likelihood of infant survival independent of household economic resources, with use of cluster-level, rather than individual-level, data. Most studies on this topic have looked only at maternal education, where the weight of evidence has indicated a link to child survival, and focused heavily on the neonatal period rather than later child mortality. Although each of these studies has contributed important knowledge to the field, crucial questions about the scope and magnitude of how both parents' education might influence child mortality have remained unanswered.

**Added value of this study**

To our knowledge, this study significantly exceeds the scale of all previous research on the subject by combining the most comprehensive systematic review of the topic to date with new primary analysis of DHS data on 3 112 474 livebirths. The systematic review was not restricted by time, location, or language, yielding 5339 individual records and involving full-text review in 15 languages. Moreover, novel mixed-effects meta-regression models were implemented here as tools to distil this extensive and heterogeneous dataset, incorporating partner's education level and household wealth or income as covariates. These models provided effect size estimates from data from 92 countries describing the relationship between parental education and childhood mortality. This study is not only the most comprehensive study on the effects of maternal education, but also significantly advances the science on how increased paternal education is associated with lower child mortality at all ages under 5 years. We highlight gaps in the field, such as the scarce research on mortality between ages 5 and 18 years, and the complexity of isolating the unique effect of parental education in the family socioeconomic context. This study is an important step towards understanding the distinct effect that education has on health generally, with child mortality examined here due to its stark and persistent global disparities.

**Implications of all the available evidence**

Over the past several decades, major international campaigns have addressed education and child survival, notably the Convention on the Rights of the Child and the UN Millennium Development Goals (MDGs). Despite impressive progress made towards the MDGs, neither its under-5 mortality goal nor the primary education goal were achieved by 2015. This unfinished agenda was further extended in the Sustainable Development Goals (SDGs), which include targets to reduce infant and child mortality (SDG 3), achieve inclusive and equitable quality education (SDG 4), and reduce inequalities (SDG 10). This study offers robust findings that can be used to mobilise evidence-based investment and encourage coordination between research, policy, and practice.

Parental education has been linked to lasting improvements in child health and life expectancy^7^, ^8^ through direct and indirect effects mediated by other determinants of health, such as socioeconomic status and living conditions.^9^, ^10^ Higher maternal education in particular has been associated with lower child mortality beyond the effect of economic and other determinants.^2^, ^11^, ^12^, ^13^, ^14^ Lohela and colleagues^9^ reported lower early neonatal mortality for the most educated mothers compared with the least educated in 72 low-income and middle-income countries, but little research has examined child mortality after the first month of life. Maternal education has generally been shown to have a stronger correlation with child mortality, compared with paternal education,^15^, ^16^ although the evidence is mixed.^17^, ^18^, ^19^ Although paternal education is associated with reduced rates of stillbirth and increased child survival,^15^, ^20^ globally, the effect of paternal education is crucially underexamined.^17^, ^21^ The lack of focus on paternal education might represent a missed opportunity to identify mechanisms that contribute to reducing under-5 mortality and narrowing health inequalities.

In striving to clarify the magnitude of the effect on maternal education on under-5 mortality, evidence for causality has been proposed.^11^, ^22^ Indeed, formal education has been suggested as a so-called social vaccine.^23^ However, study methods, populations, and study designs have been inconsistent, often using average or community-level education and mortality data. This substantial variation in study methods has made it challenging to compare across contexts and systematically account for the potential variation in effect sizes across space or time.

In this study, we aimed to investigate whether, and to what extent, parental education is a risk factor at the global level for all-cause mortality among neonates, infants, and children younger than 5 years. To provide the most comprehensive analysis to date, we aimed to exceed previous efforts in scale and geographical scope by combining global systematic review and novel primary analysis of Demographic and Health Survey (DHS) microdata.

## Methods

### Search strategy and selection criteria

For this systematic review and meta-analysis, our literature search identified articles that used individual-level data to estimate the relationship between inequalities in child mortality and parental education. Our methods were described in the protocol established before the review and registered with PROSPERO (CRD42020141731). The academic literature search took place initially between Jan 23 and Feb 8, 2019, and was updated on Jan 7, 2021, for studies published since 2019, by use of the following databases: CINAHL, Embase, MEDLINE, PsycINFO, PubMed, Scopus, and Web of Science. The search strings were designed, tested, and applied by research librarians and optimised for each database and their particular syntax. No date or language restriction was applied. The list of keywords and an example search string are available in appendix 1 (p 1).

We used Endnote, version X9.2, for the removal of duplicate records. Screening of titles, abstracts, and full text was done by teams of two reviewers independently using Excel. Discrepancies during screening were resolved by consensus or referred to a third reviewer. The reference lists of included articles were hand-searched and screened by at least one reviewer for additional references.

**Figure 1:**
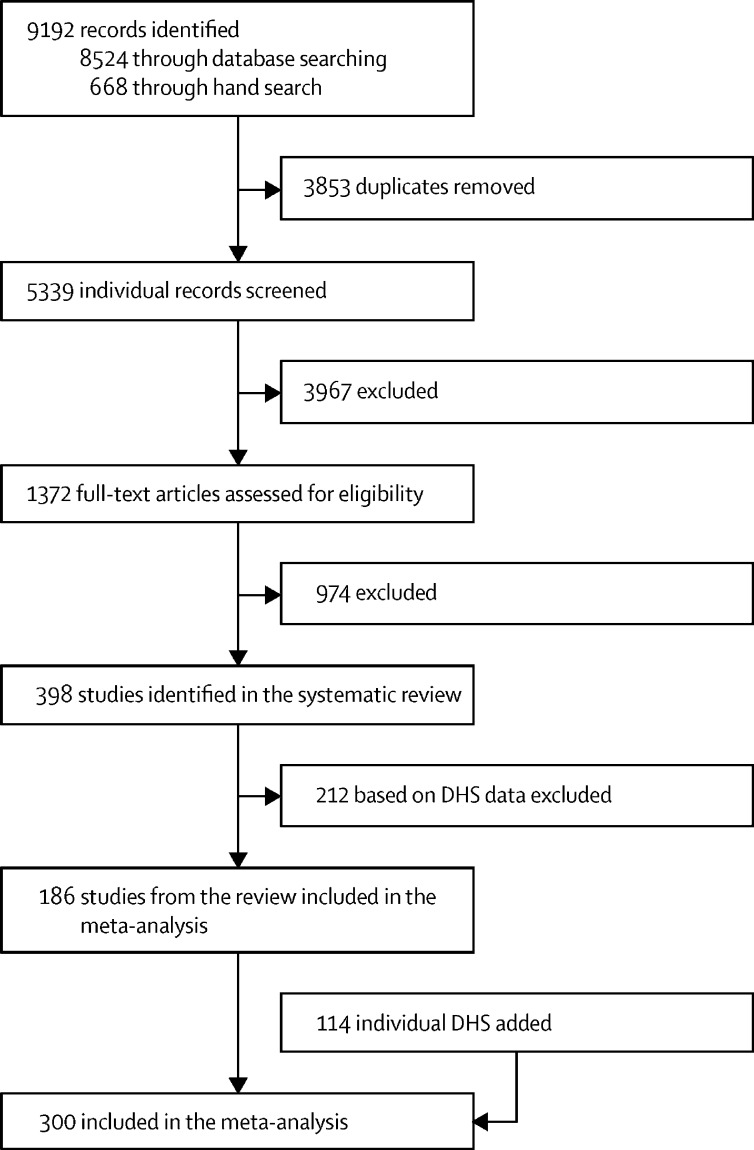
Study selection DHS=Demographic and Health Survey.

We included articles that assessed the relationship between parental education (defined as years of schooling, highest educational attainment, or literacy) and mortality of their child at any age under 5 years (table 1). Age groups were defined as neonatal (0–27 days), post-neonatal infancy (28–364 days or 1–11 months), or childhood (1–4 years). Most of the included studies were research articles that measured maternal education, while a smaller number measured paternal education or both. For non-research articles (eg, comments or letters) referring to suitable data, the relevant primary study was located and assessed for inclusion instead. Articles in any language were eligible for inclusion, and full-text screening was done in English, Spanish, French, Portuguese, Italian, Romanian, German, Norwegian, Polish, Russian, Greek, Chinese, Korean, Indonesian, and Farsi. Study selection based on PRISMA guidelines^24^ is provided in figure 1.

**Table 1:** Inclusion and exclusion criteria for systematic review

		**Inclusion criteria**	**Exclusion criteria**
Sample		No limitations based on the population sample characteristics or size	Studies not providing an accurate sample size for the relevant data
Phenomenon of interest		Study participants were children and their parents, to measure child mortality according to parental education	..
	Outcome	All-cause mortality	Cause-specific mortality; stillbirth or miscarriage alone
	Period of mortality observation	Childhood from livebirth until age 5 years	Age ≥5 years only; combined <5 years and >5 years estimates; unclear or undisclosed age group
	Measure of parental education	Literacy status; years of education; education level	Both parents' education summarised in one measure; unclear definitions of education categories; different types of education (eg, general *vs* vocational) with the same number of years
Design		Retrospective cohort; prospective cohort; cross-sectional; case-control; nested case-control; case-cohort; randomised controlled trial; non-randomised controlled trial; non-randomised trial	Case-crossover; ecological
Evaluation			
	Data	Individual level	Aggregate level; country level; rounded effect sizes; neighbourhood level alone
	Measure	Relative risk; hazard ratio; odds ratio; rate ratio	Standardised incidence ratio alone; standardised mortality ratio alone; time-to-event ratio alone; incidence alone; risk difference alone; relative index of inequality; concentration index
Research or publication type		Any academic publication (research articles, comments, editorials, reviews, letters, and so on) containing quantitative data	Studies using DHS data

Criteria are grouped on the basis of the SPIDER model (sample, phenomenon of interest, design, evaluation, and research type). DHS=Demographic and Health Survey.

Although the initial review aimed to capture child mortality until age 18 years, combined effects of parental education, and concentration index, too few studies were available to include these in the meta-analysis. Therefore, the scope was narrowed to under-5 mortality and the exclusion criteria revised to reflect this (table 1). Results based on DHS data were not extracted from published studies, because primary DHS data were used for meta-analysis instead. Included articles are listed in appendix 1 (pp 25–36).

We extracted information about the study, participants, methods, context, effect size, and covariates from each included study into a standard template from the Global Burden of Diseases, Injuries, and Risk Factors (GBD) Study, tailored to this review. The extraction template and raw extraction data are included in appendix 2. Each extraction was done by one reviewer. For each reviewer, a 10% random sample of extractions was checked by a second reviewer for quality and accuracy. If an article did not report education as a numerical value (ie, highest level of schooling completed or vocational training), we used the International Standard Classification of Education (ISCED) mapping from the UN Educational, Scientific, and Cultural Organization to determine the numerical equivalent. In the rare case that this information could not be obtained through ISCED, we obtained it through alternative sources (eg, government website or other studies). For studies that defined groups based on literacy, we used 1 to 18 years of education as the corresponding numerical value for literacy and 0 for illiteracy. Study-level risk of bias was not included in the extraction. However, studies that did not provide sufficient detail on key elements (such as sample size, population, context, or variables used in adjusted models) were excluded on the basis of poor reporting quality.

### Primary analysis of DHS data

We did primary analyses using open-access DHS microdata (individual-level records). We included all DHS that contained the complete birth history module, partner's years of schooling, and the DHS wealth variable. These criteria allowed us to control for other socioeconomic factors that could influence child health. Summary characteristics of these surveys are available in appendix 1 (p 7).

In most iterations of the DHS, female respondents of reproductive age are asked to enumerate all livebirths over the course of their lives and provide information on the survival of each child. Using these data, we constructed synthetic cohorts of children born in the years leading up to the survey date. We analysed the survival of these cohorts regarding other available characteristics, such as education, to produce estimates of differential risk of mortality by schooling (see, for example, the GBD 2016 study).^25^

We controlled for the most common study-level covariates identified in the systematic review (table 2): sex of the child, partner's years of schooling, and wealth—avoiding variables that lie on the causal pathway when possible. Detailed methods are provided in appendix 1 (pp 3–4).

**Table 2:** Summary characteristics of the systematic review

	**Outcome variable: maternal education**	**Outcome variable: paternal education**
**Number of observations**		
Total observations	1811	363
Total unique countries	64	20
Total unique studies	184	37
**Age interval**		
0–27 days	547 (30·20%)	67 (18·46%)
1–11 months	437 (24·13%)	60 (16·53%)
1–4 years	26 (1·44%)	8 (2·20%)
0–4 years	194 (10·71%)	76 (20·94%)
0–11 months	554 (30·59%)	134 (36·91%)
1 month to 4 years	53 (2·93%)	18 (4·96%)
**Study design**		
Retrospective cohort	1114 (61·51%)	218 (60·06%)
Cross-sectional	407 (22·47%)	109 (30·03%)
Prospective cohort	163 (9·00%)	29 (7·99%)
Case-control	102 (5·63%)	7 (1·93%)
Nested case-control	10 (0·55%)	0
Randomised controlled trial	7 (0·39%)	0
**Study-level characteristics**		
Representative of national or subnational unit	1207 (66·65%)	208 (57·30%)
**Studied years**		
1970–79	76 (4·20%)	27 (7·44%)
1980–89	443 (24·46%)	47 (12·95%)
1990–99	555 (30·65%)	93 (25·62%)
2000–09	500 (27·61%)	184 (50·69%)
2010–19	237 (13·09%)	12 (3·31%)
**GBD super-region**		
High-income	773 (42·68%)	206 (56·75%)
South Asia	257 (14·19%)	98 (27·00%)
Latin America and Caribbean	192 (10·60%)	1 (0·28%)
Sub-Saharan Africa	142 (7·84%)	16 (4·41%)
Central Europe, eastern Europe, and central Asia	84 (4·64%)	6 (1·65%)
Southeast Asia, east Asia, and Oceania	61 (3·37%)	5 (1·38%)
North Africa and Middle East	46 (2·54%)	15 (4·13%)
**Study-level controls**		
Controlled for age of mother	812 (44·84%)	240 (66·12%)
Controlled for sex of child	454 (25·07%)	141 (38·84%)
Controlled for wealth or income	219 (12·09%)	55 (15·15%)
Controlled for urbanicity	239 (13·20%)	41 (11·29%)
Controlled for partner's education	143 (7·90%)	135 (37·19%)
Controlled for both partner's education and wealth or income	50 (2·76%)	39 (10·74%)

Data are n (%). Percentages indicate proportion of data points with the given characteristic, displayed by parent gender. GBD=Global Burden of Diseases, Injuries, and Risk Factors Study.

### Meta-analysis combining data from systematic review and DHS primary analyses

We did mixed-effects meta-regression in this study, using the MR-BRT meta-analysis software package described by Zheng and colleagues,^26^ with technical details provided in appendix 1 (pp 37–38). We used a ratio model that allows for the integration of relative risk (RR) point estimates with different exposure and referent categories (described in Zheng and colleagues, section 2.5).^26^ Additionally, this model allows for fitting non-linear dose–response relationships where necessary, though we deemed this unnecessary for this analysis, and automated outliering of data.

We did separate meta-analyses for maternal and paternal education. Each meta-analysis incorporated study-level covariates indicating whether the associated effect size controlled for wealth, urbanicity, education, age of the mother, sex of the child, or any combination of these. Additionally, interacting covariates were included to allow the main effect to vary by the age group of the child (appendix 1 pp 2–3).

The estimated effect sizes presented here reflect adjustment for a standardised set of study-level covariates: wealth or income, partner's years of schooling, and sex of the child. 95% CIs are also reported. The estimates do not account for age of the mother at birth, which we deemed to lie on the causal pathway between parental education and child survival. Sensitivity analyses and our approach to standardising non-standard data are available in appendix 1 (pp 3–5).

### Quality and risk of bias of and across individual studies

The meta-analysis framework incorporates the uncertainty associated with each individual effect size into the model fitting process, to decrease the risk of bias introduced by study design and quality. The model, in turn, estimates the degree of between-study heterogeneity, or γ (appendix 1 pp 37–38). To assess the risk of bias across studies, we applied funnel plots to the residuals of each model, to visually inspect how individual study effect sizes deviate from the average fit. Each point was plotted by use of the residual value on the x-axis and reported SEs on the y-axis, with points falling within the funnel consistent with reported uncertainty, with random effect units being per year of education in log space. Additionally, we report the square root of γ, or the SD of between-study heterogeneity, as an empirical measure of the level of heterogeneity observed.

### Role of the funding sources

The funders had no role in study design, data collection, data analysis, data interpretation, or writing of the report.

## Results

Of the initial 5339 individual records captured by our systematic review search, 398 matched our inclusion criteria. Of these, 212 DHS-based articles were excluded. A total of 186 studies from the systematic review together with 114 unique DHS surveys (yielded a combined total of 300 studies, published between 1982 and 2020 from 92 countries, for inclusion in the meta-analysis (figure 1). Extracted effect sizes were transformed to match model-specification, age-interval, or parent's gender combinations, resulting in 2174 extracted effect sizes suitable for analysis.

**Figure 2:**
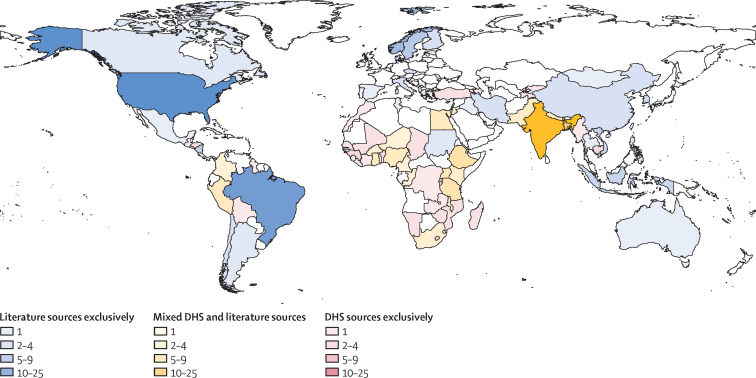
Mapping of included studies by location and data type This map shows the number of unique sources identified and extracted from the systematic review across all age ranges for each geographical unit. Studies that represented subnational units or cities are mapped here to their parent countries. Colour indicates the type of data source used in the meta-analysis by country, with darker colours indicating a greater number of unique data sources. DHS=Demographic and Health Survey.

Although all included studies were published in the past 40 years, they covered a wider timespan, with cohorts starting as early as 1967, and cross-sectional studies covering the lifetimes of the mothers interviewed. The literature was biased heavily towards studies on the link between child survival and maternal education (1811 [83·3%] of 2174 observations), rather than paternal education (363 [16·7%]). The high-income super-region (as defined by the GBD Study)^27^ accounted for 45·0% of observations used in the meta-analysis (figure 2).

Of the studies included in the systematic review, only five covered both children younger than 5 years and older children, whereas four included exclusively late childhood and adolescence and were excluded because of low power for this age interval (table 2). A minority of studies disaggregated their results by age intervals, with only five analysing the age range of 1–4 years. Data quality varied substantially by study. A wide range of study-level covariates were included, with some lying on the causal pathways between parental education and child mortality. The most common study-level confounders found in included primary studies were child sex, mother's age at delivery, wealth or income, rural or urban residence, and partner's education. Only 89 (4·1%) of observations met our criteria for appropriately controlled effect sizes (ie, controlling for the other parent's education and wealth or income). A total of 1415 (65·1%) of observations came from country-wide samples. Five studies provided dose–response estimates for the effect of parental education on child mortality.

DHS data from 114 unique surveys across 58 countries were used for primary microdata analysis and combined with extracted systematic review data for separate meta-analyses for maternal and paternal education. These individual-level DHS data were drawn from 875 396 mothers and captured 3 112 474 livebirths and 318 619 deaths of children younger than 5 years. This allowed examination of parental education on a continuous scale and child mortality within discrete age intervals. Sensitivity analyses indicated no compositional biases by data source (appendix 1 pp 23–24). Although some of the estimates of paternal education's effects on child mortality using systematic review data alone seem to indicate a deleterious effect of paternal education on child survival, they are insignificant findings.

### Maternal and paternal education and their relationship with under-5 mortality

**Figure 3:**
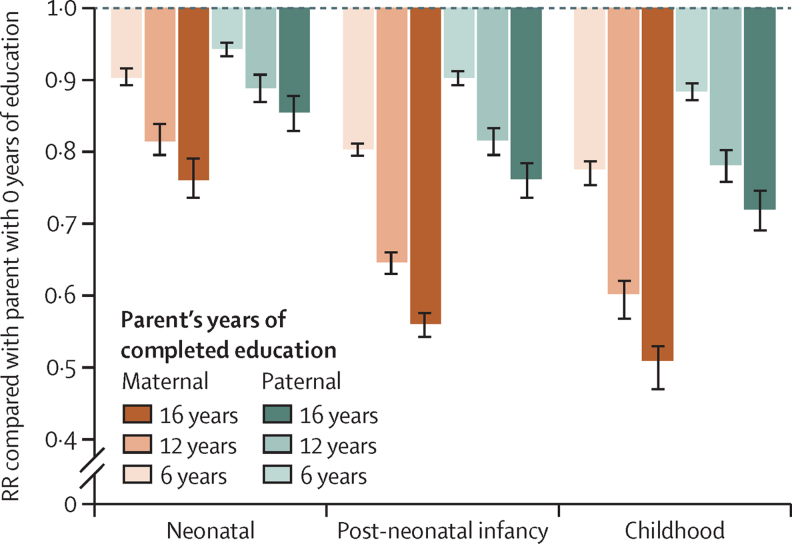
Summary of RRs of child mortality by parental education Error bars are 95% CIs. RRs of child mortality are shown for three age intervals: neonatal (1–27 days), post-neonatal infancy (1–11 months), and under-5 childhood (1–4 years). Maternal education and paternal education are shown by completed years of schooling (colours darken with increasing years of education). All levels of parental education were compared with 0 years of education as reference level. RR=relative risk.

**Figure 4:**
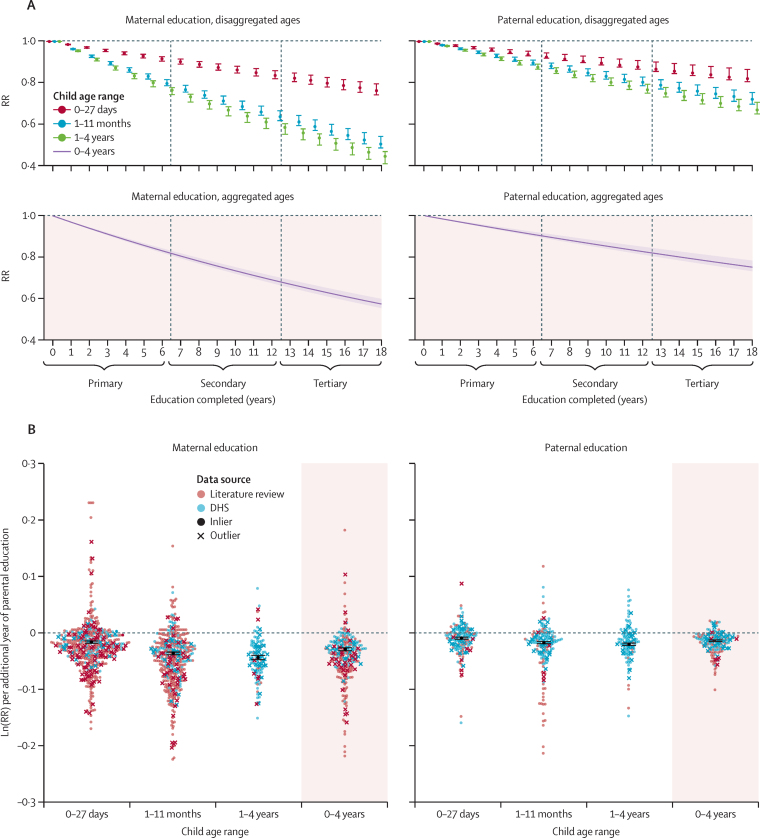
RR of under-5 mortality by parent's education (maternal and paternal) and child age (A) These RR curves show fitted average effect sizes in normal space across the full range (0–18 years of parental education) of exposures. (B) This figure shows how the underlying, normalised data were synthesised to produce the RR curves; normalised ln(RR) can be interpreted as the instantaneous slope of the RR curve implied by each study; data are superimposed with a synthesised average effect size; all of this is done separately by age group, and the other age groups estimated in the model are presented in appendix 1 (p 9). DHS=Demographic and Health Survey. RR=relative risk.

Figure 3 presents a summary of relative risks of child mortality in the neonatal period, infancy, and childhood, by parental completion of primary, secondary, and tertiary education. Each additional year of maternal education, compared with a mother with no education, was associated on average with a reduction in mortality for children younger than 5 years of 3·0% (95% CI 2·8–3·2). Compared with a mother with 0 years of education, this finding translates to a reduction in mortality of 16·9% (95% CI 15·8–17·9) for children younger than 5 years born to mothers with 6 years of education (ie, completed primary education), 31·0% (29·0–32·6) for children born to mothers with 12 years of education (ie, completed secondary education), and 39·0% (36·7–40·9) for children born to mothers with 16 years of education (ie, completed 4-year tertiary education; figure 4).

Paternal education showed a similar, but smaller effect compared with maternal education. Each additional year of paternal education was associated on average with a reduction in mortality for children younger than 5 years of 1·6% (1·3–1·7). Compared with a father with 0 years of education, this finding translates to a reduction in mortality of 9·1% (7·8–9·9) for children younger than 5 years born to fathers with 6 years of education, 17·3% (15·0–18·8) for children born to fathers with 12 years of education, and 22·4% (19·5–24·2) for children born to fathers with 16 years of education (figure 4). Sensitivity analyses (appendix 1 pp 4–5) showed that incorporating additional study-level covariates beyond sex of the child, partner's years of schooling, and wealth did not have a large effect on our final estimates.

Most extracted study effect sizes (69·8%) found a significant and protective effect of maternal education on under-5 survival (appendix 1 p 19). Considerably fewer effect sizes (53·7%) found significant and protective effects between paternal education and under-5 survival (appendix 1 p 13).

### Disaggregating under-5 mortality by age

**Figure 5:**
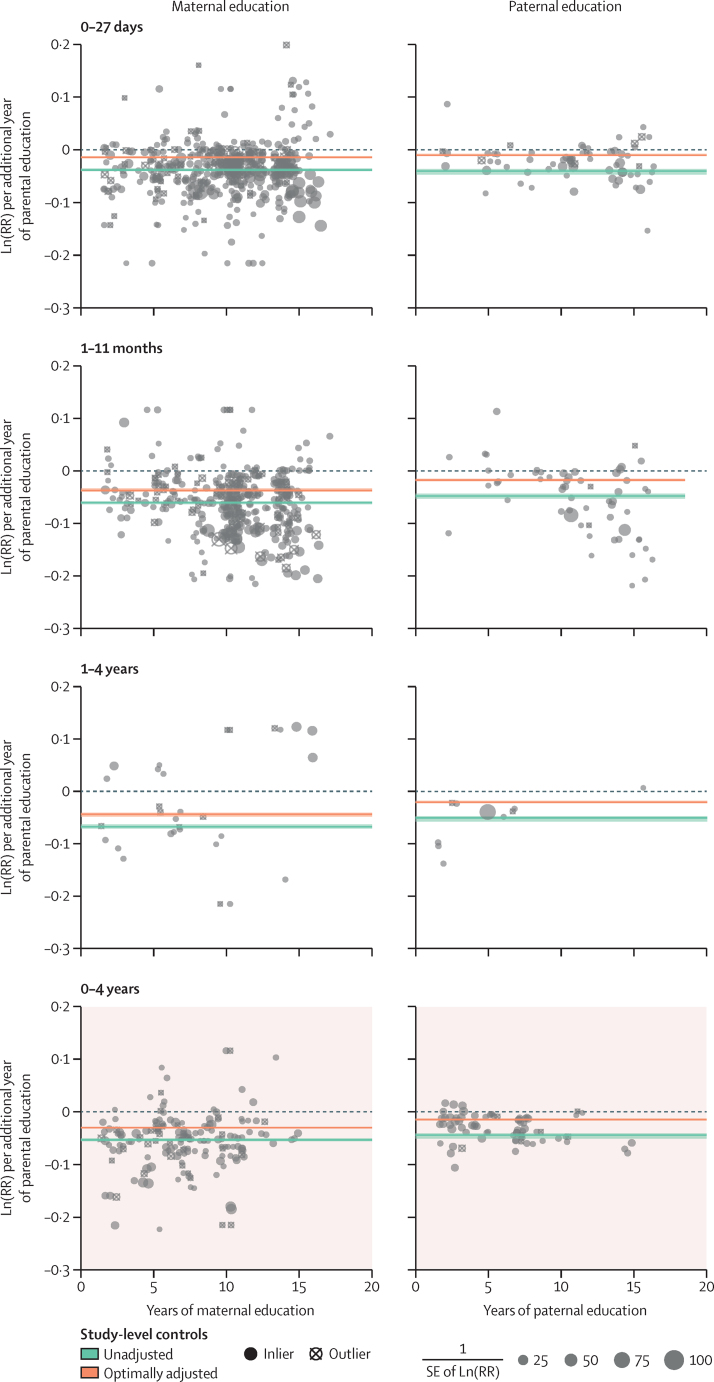
Dose–response relationship between parental education and child mortality By displaying the data from figure 4B across the entire exposure range, we are able to examine the monotonicity and linearity of the data. Models are adjusted for wealth or income, the partner's level of education, and sex of the child. RR=relative risk.

Compared with a mother with no education, each additional year of maternal education resulted, on average, in a reduction in mortality for neonates (0–27 days) of 1·5% (1·3–1·6). For post-neonatal infants (1–11 months or 28–364 days), the reduction was 3·7% (3·3–3·9), and for young children (12–59 months or 1–4 years), the reduction was 4·4% (4·1–4·8; figure 4). This resulted in a child born to a mother with 12 years of education, compared with one born to a mother with no education, having a 16·4% (14·2–18·0) reduced risk of dying in the first month of life, a 36·3% (33·5–38·2) reduced risk of dying between 1 and 11 months, and a 41·5% (39·7–44·8) reduced risk of dying between 12 and 59 months (figure 4). Similarly, each additional year of paternal education resulted in a reduction of mortality for neonates (0–27 days) of 1·1% (0·8–1·2) compared with those born to a father with no education. For post-neonatal infants (1–11 months or 28–364 days), this reduction was 1·8% (1·6–2·0), and for young children (12–59 months or 1–4 years), the reduction was 2·2% (1·9–2·4; figure 2). This resulted in a child born to a father with 12 years of education, compared with one born to a father with no education, having a 12·3% (9·2–13·4) reduced risk of dying in the first month of life, a 19·6% (17·2–21·4) reduced risk of dying between 1 and 11 months, and a 23·3% (20·7–25·0) reduced risk of dying between the ages of 1–4 years (figure 4). Figure 5 shows the dose–response relationship observed between parental education and under-5 mortality for all age intervals and both parents. The slope of the RR curve was negative across the entire exposure range. This offered, in aggregate, no evidence for a decreasing marginal utility of increased maternal or paternal education (more details in appendix 1 p 6).

### Effect sizes across time and geography

**Figure 6:**
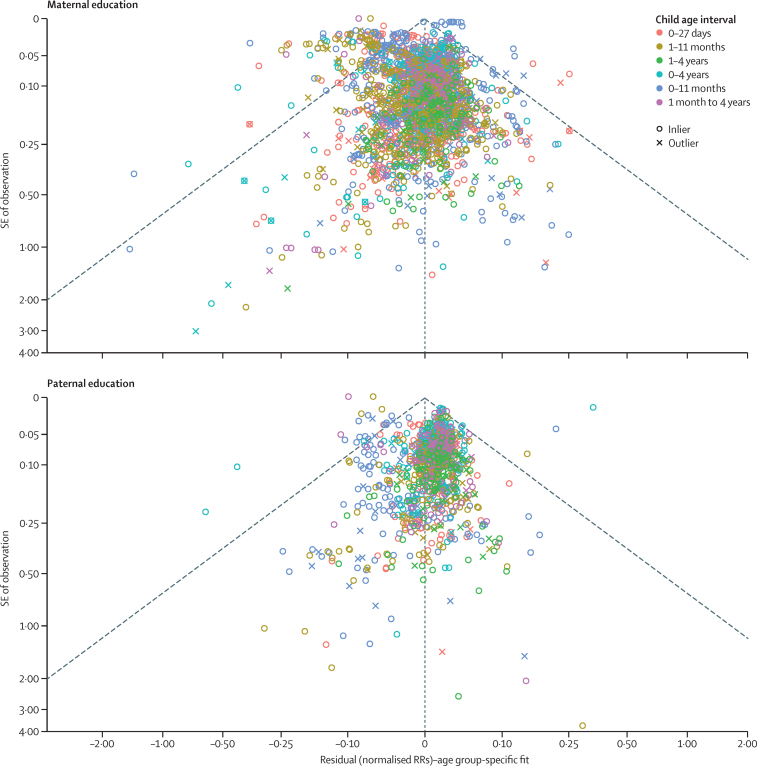
Funnel plots of effect sizes extracted in the systematic review Funnel plots show how the effect sizes of RRs from individual studies systematically vary according to the SE of their observations. Because each child age interval has a different average effect size, as estimated by our models, we plotted the residuals against the SE of the observations. The residuals are defined as the normalised RR of the study minus the age-specific fit. Many studies outside of the funnel would indicate study-level heterogeneity and indicate more deviation from the average effect size than would be expected from chance alone. RR=relative risk.

Figure 6 shows that few data points fell outside the funnel of estimated average effect sizes. This was corroborated by the between-study random effects SD estimation in our model (table 3). We assumed that between-study heterogeneity translates roughly to between-geography heterogeneity, an assumption that was strengthened by our sensitivity analysis, which showed rather consistent results between DHS and non-DHS data. The maternal model returned an SD of 0·0115% (95% CI 0·0105–0·0124) and the paternal an SD of 0·0067% (0·0058–0·0076). These values translate to an absolute average magnitude of deviation of the study-specific log (RR) from the model fit of a factor of ±0·138% (95% CI 0·126–0·149) for the maternal model and ±0·080% (0·070–0·092) for the paternal model. As an example, we estimated that the average reduction in risk of a child dying in their first 5 years of life for a mother with 12 years of education compared with 0 years of education to be 31·0%. Adding study-level uncertainty to these estimates gives the range of possible RRs to be between 39·0% and 23·0%.

**Table 3:** Coefficients from meta-analyses

	**Paternal coefficients**	**Maternal coefficients**
**Exposure**		
Education, years	−0·046 (−0·051 to −0·042)	−0·054 (−0·057 to −0·050)
**Study-level covariates**		
Rural or urban:education	0·001 (−0·004 to 0·006)	0·007 (−0·003 to 0·012)
Wealth or income:education	0·023 (−0·018 to 0·030)	−0·007 (−0·012 to −0·001)
Partner's education:education	0·006 (0·001 to 0·011)	0·011 (0·006 to 0·014)
Child sex:education	0·001 (−0·006 to 0·009)	0·011 (0·007 to 0·015)
Mother's age:education	0·010 (0·007 to 0·013)	0·007 (0·005 to 0·010)
**Child age dummy variables**		
0–27 days:education	0·005 (0·003 to 0·007)	0·013 (0·011 to 0·014)
0–11 months:education	0·002 (−0·000 to 0·003)	0·003 (0·001 to 0·004)
1–11 months:education	−0·003 (−0·005 to −0·001)	−0·008 (−0·009 to −0·006)
1 month to 4 years:education	−0·005 (−0·006 to −0·003)	−0·008 (−0·010 to −0·006)
1–4 years:education	−0·006 (−0·009 to −0·004)	−0·015 (−0·018 to −0·012)
**Between-study heterogeneity**		
SD per year of education	0·007 (0·006 to 0·007)	0·011 (0·010 to 0·012)

The reference child age dummy variable (not shown) is 0–4 years (under-5). Parental education was modelled as a continuous variable, and all interactive variables are operationalised as such. All variables aside from the main exposure are binary variables that interact with continuous education and capture study-level qualities alone.

## Discussion

To our knowledge, our global systematic review and meta-analysis provides the most comprehensive synthesis to date of evidence describing the relationship between parental education and inequalities in all-cause child mortality from livebirth to 5 years of age. The results show that lower maternal and paternal education are both risk factors for under-5 mortality at all ages, even after controlling for wealth or income, partner's years of schooling, and sex of the child.

Meta-analyses showed that both increased paternal and maternal education were linked to reduced all-cause child mortality globally. A child born to a mother with a high-school degree had a 31·0% (95% CI 29·0–32·6) lower risk of dying before their fifth birthday than one born to a mother with no education. This was supported by findings reported for maternal education in several low-income and middle-income countries.^9^, ^13^, ^28^ Meanwhile, a child born to a father with a high-school degree had a 17·3% (15·0–18·8) lower risk of dying before their fifth birthday than one born to a father with no education. Disaggregation by child age indicated that the protective effect of each parent's education was significant across all age groups and grew stronger with increasing age of the child. Across age groups, maternal education was a stronger predictor of mortality than was paternal education. Moreover, the systematic review found evidence of this relationship across world regions. Although variations in effect sizes existed among the reviewed studies, this variation was not in excess of what would be expected by chance, indicating a moderately consistent relationship between parental education and the prevention of child mortality across time and space.

Globally, most child deaths occur during infancy,^29^, ^30^ with the first 28 days of life being the most vulnerable period for mortality due to prematurity, congenital anomalies, and pregnancy complications.^31^, ^32^, ^33^ Progress in reducing neonatal mortality globally has been slower than that for older children, leading to a growing share of neonates among under-5 deaths.^34^, ^35^, ^36^ We report 1·5% (95% CI 1·3–1·6) lower neonatal mortality per year of education for mothers, and 1·1% (0·8–1·2) for fathers. Because neonatal causes of death are strongly influenced by antenatal and perinatal health-care quality and access, it is not surprising that the relationship between neonatal mortality and parental education was smaller than that for post-neonatal infants and children. Still, even a comparatively small reduction of neonatal mortality linked to an additional year of maternal education in relative terms might, in fact, contribute to a substantial number of babies' lives saved in absolute terms.

We showed that the link between both paternal and maternal education and child survival is strong, highlighting the intergenerational effect on health conveyed by increased education, probably through several mechanistic pathways. Explanatory factors or mediators might include parents' health literacy,^37^ health-seeking behaviours,^38^, ^39^ consanguinity and family structure,^40^, ^41^ and quality early care and education.^42^ Education also plays a key moderating role between health interventions and child health determinants and outcomes.^43^ The attenuated effect of paternal education might reflect the gendered pathways through which parental education affects child mortality. The scarce research on the combined effects of maternal and paternal education suggests that effects of both parents' education, together with assortative mating and community education level, might all play a role.^17^, ^44^ The persistent relationship between maternal education and child mortality points to gender-specific pathways,^45^ such as increased female autonomy, resources, and knowledge that might translate into improvements such as increased use of health services and health-seeking behaviours,^2^, ^46^, ^47^, ^48^, ^49^, ^50^, ^51^ delivery in health facilities,^52^ greater autonomy in deciding parity levels and reducing fertility,^45^, ^53^ and better child nutrition.^54^ Moreover, maternal education and literacy can improve agency to influence family and child-care decisions.^55^, ^56^

This complex web of socioeconomic factors and behaviours that lies between parental education and child health necessitates careful consideration of study design.^10^, ^57^, ^58^, ^59^ For example, wealth and income are both determinants and outcomes of education. Smoking, alcohol use, antenatal care use, and age of the mother at birth also lie on the causal pathway between parental education and childhood mortality, yet are commonly used as study-level covariates in the literature.^60^, ^61^ Additionally, included studies used a wide range of exposure and referent categories (eg, reference category of 0 years of education, some high school, and so on), had inconsistent use of a continuous exposure measure for parental education, and often reported multiple relevant effect sizes in a single study. To address the methodological challenge posed by the diversity of study designs and analytical approaches yielded by the systematic review and the synthesis with DHS microdata, we implemented a novel ratio model using the MR-BRT meta-regression tool, to integrate RR point estimates with different exposure and referent categories. All models were adjusted for wealth or income, partner's education, and sex of the child. By striving to isolate the relationship between parental education and child mortality, this study can aid future research in measuring the effect of other markers of socioeconomic inequalities and improving comparability across contexts. Previous studies have estimated that each year of increase in average maternal education was associated with reductions in under-5 mortality by 8·5%^21^ or 9·5%,^13^ though these analyses relied on country-level indicators that lacked specificity about individual families' socioeconomic status. We found, when using individual-level microdata and—unlike previous studies—also controlling for partner's education, that these effects are attenuated, with each additional year of maternal education associated with an under-5 mortality reduction of 3·0% (95% CI 2·8–3·2). A comprehensive analysis of both parents' education alongside other determinants of health such as welfare, living environment, sanitation, food, working conditions, and access to quality health and social services, could help translate research to inform the development of interventions for improving child health outcomes through the social determinants of health.^62^

Unlike previous research drawing on data from limited geographical contexts,^63^, ^64^, ^65^ our meta-analysis supports a linear dose–response relationship between increased parental education and lower child mortality at the global level. We found no evidence that the first years of education were more strongly predictive of child survival than later years, consistent with the sparse existing research base.^66^, ^67^ This review provides novel evidence of the same age trend being present among fathers. The dose–response relationship serves to highlight the importance of universal primary school completion—part of the unfinished development agenda the SDGs sought to address—in equal step to the importance of higher education as it pertains to health. Despite this, little progress has been made towards achieving universal higher education.^68^

The findings presented here should be interpreted while accounting for the study's limitations, as well as key gaps in the field. First, this study reports the average global relationship between parental education and all-cause child mortality, the generalisability of which was supported by the data in aggregate and in sensitivity analyses. However, more granular effects, such as those specific to a location or time, also warrant careful consideration. Second, methodological choices—such as the age intervals used, decision to examine maternal and paternal education separately, and selection of covariates—were strongly influenced by what was available from the data found in the literature. For example, too few published studies covering child mortality after age 5 years and in adolescence were available to be included in this study. Moreover, many studies did not adequately disaggregate by child age despite clear changes to the disease profile of child health throughout the first 5 years of life. We used DHS data to fill this gap, which is particularly relevant for the under-studied period of 1–4 years, which has a relatively high mortality rate in sub-Saharan Africa compared with other regions.^69^ Third, the systematic review results reflect longstanding imbalances in the field, with disproportionately more studies from high-income countries and fewer data sources from low-income and middle-income countries outside of DHS-based studies, as well as a large bias towards studying maternal, rather than paternal, education.^16^ Fourth, few observations obtained from our review adequately controlled for at least two key social determinants of health: the other parent's education and an indicator of wealth or income. The meta-analyses addressed this by controlling for wealth or income, partner's educational attainment, and sex of the child for calculating effect sizes, to harmonise varying methods across data sources and indicate the strength of effects accounting for family socioeconomic status. Fifth, this study covers a period spanning several decades that witnessed large reductions in child mortality. Over the past 50 years, vaccination campaigns, improvements in antenatal care, and interventions against communicable diseases including malaria, lower respiratory infections, diarrhoeal diseases, and measles have boosted child survival in low-income and middle-income countries.^70^, ^71^ At the same time, child mortality trends in high-income countries not only reflect social policies such as expanded health-care coverage, but also widening social inequalities in maternal health.^72^ Further research is needed to disentangle the interacting effects on child mortality of parental education and trends in health-care access and quality. Finally, the study leaves open questions about the relationships between parental education and cause-specific child mortality, as well as how child mortality is related to parental preschool education, quality rather than years of education, or combined effects reflecting both parents' education levels.

Education offers a key point of entry globally to improve the health of future generations and promote sustainable development through improving opportunities and participation and providing knock-on effects for other social determinants of health. To date, this is the largest study on the relationship between parental education and child mortality, providing the most comprehensive evidence quantifying the degree to which lower education of mothers and fathers is a risk factor for under-5 mortality. The reduction in the RRs of under-5 mortality brought by each additional year of parental education observed globally in this study provides strong evidence to further support goal 4 of the SDGs, universal quality education, as a mechanism to achieve SDG target 3·2 of reducing neonatal and child mortality. This study represents an important step towards expanding the frame of reference for social determinants of health^73^ and advances our understanding of the transgenerational effect of parents' education on child mortality within the family socioeconomic context. Overall, the findings provide a strong rationale for the global development community to focus on education—starting early and continuing into higher education—on a global scale for its potential health-protective benefits on child survival. This is particularly relevant for the estimated 750 million adults who lack basic reading and writing skills, two-thirds of whom are women.^74^ This study offers robust findings that can be used to mobilise evidence-based investment to address health equity for the next generation, towards universal education and elimination of the gender gap in schools.

## Data sharing

Supplementary data files contain all raw tabulated data from the systematic review and collapsed DHS data (appendix 3); key to study names is provided in appendix 4. This study used DHS data that are available from public online repositories, most of which require a basic registration process and usage agreement with the data provider. Although we are restricted from providing the DHS data directly in most cases, specific datasets can be made available by request and with permission from the data provider. We can be contacted for assistance in acquiring data for replication of this study.

## Declaration of interests

We declare no competing interests.
