# Supplementary material for: Parental education and inequalities in child mortality: a global systematic review and meta-analysis
Source: Lancet. 2021 Aug 14;398(10300):608–20. doi: 10.1016/S0140-6736(21)00534-1 (PMC8363948; doi:10.1016/S0140-6736(21)00534-1)
Supplement: Supplementary appendix 1 [file mmc1.pdf]

THE LANCET

Supplementary appendix

This appendix formed part of the original submission and has been peer reviewed. We post it as supplied by the authors.

Supplement to: Balaj M, York HW, Sripada K, et al. Parental education and inequalities in child mortality: a global systematic review and meta-analysis. *Lancet* 2021; published online June 10. http://dx.doi.org/10.1016/S0140-6736(21)00534-1.

Supplementary Appendix I

Supplementary Methods

1. Sample search string

The following keywords were provided to the research librarian by the review coordinator and adapted to each database:

> Infant mortality, neonatal mortality, child mortality, under-5 mortality, under-15 mortality, under-five mortality, under-fifteen mortality, under five mortality, under-fifteen mortality
>
> Educational status, educational attainment, maternal education, parent's education, parental education, mother's education, socio-economic status, socioeconomic status, parental socio-economic status, parent's socio-economic status, mother's socio-economic status, father's socio-economic status, parent's socioeconomic status, mother's socioeconomic status, father's socioeconomic status, maternal socioeconomic status, paternal socioeconomic status, parental socioeconomic status, social class.

Database: Web of Science

Date of the search: 04.02.2019

> TS=("Infant mortality" or "neonatal mortality" or "child mortality" or "under-5 mortality" or "under-15 mortality" or" under-five mortality" or "under-fifteen mortality" or "under five mortality" or "under-fifteen mortality")
>
> TS=("Educational status" or "educational attainment" or "maternal education" or "parent* education" or "mother* education" or "socio-economic status" or "socioeconomic status" or "parent* socio-economic status" or "mother* socio-economic status" or "father* socio-economic status" or "parent* socioeconomic status" or "mother* socioeconomic status" or "father* socioeconomic status" or "maternal socioeconomic status" or "social class" or SES)

These are combined using the "Combine sets" function in Web of Science with the AND Boolean operator.

2. Equations

Equation 1. For interpretability purposes of the model coefficients, we standardized the DHS wealth variable to lie between 0 and 1 using the equation below:

$$I_{hh,i} = \frac{W_{hh,i} + \min(W_{hh})}{\max(W_{hh}) - \min(W_{hh})}$$

Where:

- $I_{hh,i}$ is the standardized wealth metric for household i, scaled to lie between 0 and 1, and
- W_{hh} is a vector of all wealth metrics measurements for a given survey, and
- $W_{hh,i}$ is the unstandardized wealth metric for household i.

Equation 2. For each survey-year-age-span, we ran a Cox proportional hazards model with random effects (frailty model), operationalized as follows:

$$\boldsymbol{ln\left(\frac{\lambda(t)}{\lambda_0(t)}\right) = \beta_1 * Maternal\ Education + \beta_2 * Partner's\ Education + \beta_3 * DHS\ Wealth\ Index + \beta_4 * Year\ of\ Birth + \beta_5 * Child\ Sex + \beta_6 * Maternal\ Stunting + \alpha_1 + \gamma_1}$$

Where:

- $\lambda(t)$ is the hazard function at time = t,
- $\lambda_0(t)$ is the baseline hazard function at time = t,
- *Maternal Education* is years of education of the mother,
- *Partner's Education* is years of education of the partner (usually the father),
- *DHS Wealth Index* is the continuous DHS wealth index (standardized to lie between 0 and 1),
- *Year of Birth* is the year of birth of the child centered by subtracting 1980,
- *Child Sex* is a binary indicator capturing whether the child is male or female, and
- α_1 is a normally distributed random effect on the first administrative unit,
- and γ_1 is a normally distributed nested random effect on the mother's birth cohort (in decades).

Resulting continuous relative risks corresponding to e^{β_1} or e^{β_2} for the relative risk of maternal or paternal education per one year of education were then used as data points in the meta-analysis.

Equation 3. For the meta-analysis, we included random intercepts for studies, so that multiple effect sizes from the same study did not drive the results unnecessarily. Outlier trimming was set at 10%. The model took the following form:

$$y = (X_{alt} - X_{ref}) \times (X_{cov}\beta_{cov} + \beta + u)$$

Where:

- y is the log(relative risk),
- X_{alt} and X_{ref} are midpoints for alternative and reference intervals for the exposed and unexposed groups measured in the relative risk effect size
- X_{cov} is a design matrix containing covariates we control for (wealth, urbanicity education, age of the mother, sex and age bin of the child)
- β_{cov} are covariate multipliers associated with X_{cov}
- β gives estimate of effect size (effect of unit of education on log relative risk).
- u is a study-specific random effect.

More information on this model is detailed in Supplementary Appendix II.

3. Displaying Nonstandard Data

For data display purposes, in some of the following figures we represent each effect size as the relative risk or log-space relative risk of under-5 mortality per year of maternal/paternal education. This is necessary as the included studies had inconsistent referent exposure categories and were thus incomparable when viewed in normal, unadjusted space (i.e., one study may report a relative risk with respect to 0 years of education while another may report a relative risk with respect to completed secondary education). This is concurrent with the above treatment of the data by the model if the final model is linear. The abbreviated method for normalizing this data for visualization purposes divides the effect size by the distance between the midpoints of the referent and alternate exposure windows as follows:

$$\frac{Log(RR)}{Year\ of\ Schooling} = \frac{Log(RR)}{(ref_{lower} + ref_{upper} + alt_{lower} + alt_{upper})/4}$$

4. Primary Analyses of DHS Data

As discussed in the main text, we ran independent analyses for n=114 unique DHS surveys across n=58 countries. For each survey, we ran independent models across different age ranges to assess the protective effects of parental education on child survival. We controlled for the most common confounding variables identified in the systematic review – sex of the child, partner's educational attainment, and wealth –, avoiding variables that lie on the causal pathway when possible. Sensitivity analyses discussed below, however, show that incorporating additional the choices of confounders beyond these three did not have a large impact on our final estimates. These confounders included sex of the child, the partner's educational attainment, and wealth.

The DHS wealth indicator, an asset-based indicator that is country- and survey- specific, was used as a proxy for household wealth. See Equation 1 above, for standardization of the DHS wealth variable between surveys.

Finally, we also included year of birth as a continuous fixed effect variable to control for agnostic gains in child survival due to the widespread economic development that occurred during this period that varied across countries. We also included nested random effects on level 1 administrative units and the mothers' birth cohort (in decades) to further absorb agnostic average changes in child mortality in due to changing location-based characteristics over time.

For each DHS survey, we ran a set of models which corresponded to the six major under-5 age intervals found in the systematic review. These models never pooled data from different locations or survey years unlike other analyses. The reasons for this are threefold. First, by limiting our reference frame to a single survey iteration, we allow comparability in effect size estimates across countries. If we instead pooled data across locations or within locations, several countries would benefit from effectively increased sample sizes resulting from multiple survey iterations while others would not. Second, by treating each survey iteration as a separate sample, we allow for comparisons within countries across time. Third, by effectively stratifying our dataset by survey iteration, we tacitly control for time-varying contextual factors such as political changes, foreign aid, and development that can change between surveys.
We ran the models identically over six different age groups, four of which are roughly analogous to the most common age bins found in the systematic literature, as discussed below.
For each survey-year-age-span, we ran a Cox proportional hazards model with random effects (frailty models), described in Equation 2 above. Resulting continuous relative risks corresponding to the relative risk of maternal or paternal education per one year of education were then used as data points in the meta-analysis.

5. Sensitivity Analysis I: Demographic and Health Survey Microdata Primary Analyses

As stated in the main paper, numerous studies were identified and excluded because they used Demographic and Health Survey (DHS) data in their analyses. We reasoned that, though our own primary analyses, we could standardize these studies to reduce noise due to variation in study design. However, there remained the question of what means was the best by which to analyze the data. As stated in the paper, we opted for survey-specific models as opposed to pooled models. These models were run separately for each survey and the effect sizes gleaned from them were used in our analysis. All models took the form of a mixed effects Cox proportional hazards model. The models operationalized maternal and paternal education as continuous variables. Included as fixed effects covariates were the DHS wealth index (standardized as explained in the main text), sex of the child, year of birth, and maternal stunting. In order to test the differential impact each of these has on the final effect sizes (the beta coefficients corresponding to maternal and paternal education), we conducted a sensitivity analysis that systematically measured their impact on the model. All models included maternal education and paternal education as fixed effects. We then tested nine other model specifications, one model

that included all covariates, four that measured the impact of adding each covariate separately, four that measured the effect of removing each covariate from the fully controlled model. Only the beta coefficients for the main effects, maternal and paternal education, were of interest. See Supplementary Figure 15.

6. Sensitivity Analysis II: Systematic Review + DHS Synthesis and Meta-Analysis

The meta-analyses we undertook synthesized data from a systematic review and Demographic and Health Surveys microdata. Owing to the nature of these two data sources, we used a sensitivity analysis to see if any compositional biases in these data adversely affected our results. The meta-analytical models mentioned in the main text of this article were rerun using three data specifications: (1) the original data that combines both systematic review data and results from our own microdata analyses, (2) only the results from the DHS microdata analyses, and (3) only the input data identified by the systematic review. In our main text, we describe several sources that were identified in the systematic review that were the result of analyses of DHS data by other parties. As we describe in the main text, all three sensitivity analyses scenarios continued not to use those data. In the case of scenario 2, which only uses results from our analyses of DHS microdata, the metaanalysis is run with no covariates. This is because there is no need to adjust for study-level covariates because all studies in this meta-analytical task are already optimally controlled. A model specification that included these covariates would have no contrast in the covariate values across the rows of data, resulting in a failed model. To judge to comparability of these scenarios, we compare the same quantities displayed in Figure 4b of the main text, notably, the logged relative risk per each additional year of paternal or maternal education across multiple age ranges. See Supplementary Figure 16.

7. Supplementary Results, Tables & Figures

Supplementary Figure 1 shows the normalized effect sizes extracted from the systematic review and the midpoints of the exposure and referent categories for each extracted effect size as an approximation of the instantaneous slope of the relative risk curve implied by each extracted effect size. This is used as a means of examining the linearity of the dose-response relationship between parental education and under-5 mortality when superimposed with the aforementioned average effect sizes. While such a task is complicated by each datum having been approximated with a different set of confounders, visual inspection is still possible so as to determine the linearity and monotonicity of the fitted relative risk curve. Across all age intervals and both parent genders, linearity of the slopes of the effect sizes is apparent.

Additionally, Supplementary Figure 1 provides evidence for the monotonicity of the dose-response relationship between parental education and child mortality (i.e. the slope of the relative risk curve is negative across the entire exposure range). While some studies have shown significant payoffs of primary schooling compared to no schooling but have shown insignificant effects of secondary schooling as compared to primary schooling (citations pulled from systematic review), these results compel us to believe that there is, in aggregate, no evidence for a decreasing marginal utility of increased maternal or paternal education.

Supplementary Figures 3 through 14 display normalized relative risks per one year of parental education, shown for all study data separately by parent's sex, child's age, and systematic review or DHS data. Color indicates inlier/outlier. Full study titles are provided in the supplementary spreadsheet. Axis labels are intentionally small. These figures serve two purposes, one of which is to simply provide an overview of data availability and effect sizes. The second is to allow curious readers to cross reference specific studies to see how their measured effect sizes compare to other studies' effect sizes.

Supplementary Table 1

Survey characteristics for DHS data

Live Births and Deaths by Country

Location	Number of Surveys	Total Mothers	Total Live Births	Deaths 0-1 Month	Deaths 1-12 Months	Deaths 12-60 Months
Albania	2	11,725	28,528	298	305	69
Armenia	2	5,422	11,546	242	192	51
Azerbaijan	1	5,001	12,936	422	431	91
Bangladesh	4	51,301	151,308	8,936	3,360	3,390
Benin	3	27,467	114,279	4,408	4,060	4,651
Bolivia	1	10,906	41,882	2,232	1,595	1,100
Burkina Faso	2	15,332	67,133	3,268	3,972	4,538
Burundi	1	2,766	11,724	549	523	581
Cambodia	3	18,543	59,322	2,663	2,096	1,061
Cameroon	2	8,204	33,086	1,267	1,418	1,536
Chad	2	12,031	56,917	2,253	2,732	3,196
Colombia	2	51,697	142,927	2,548	1,651	717
Comoros	1	2,671	10,565	314	134	105
Congo (Brazzaville)	2	7,783	28,147	896	990	884
Côte d'Ivoire	1	2,949	12,121	664	516	404
DR Congo	1	3,083	13,157	567	788	617
Egypt	3	51,618	167,847	5,036	3,928	1,540
Ethiopia	3	23,130	97,912	5,302	3,981	3,861
Ghana	3	9,332	35,295	1,508	1,225	1,069
Guatemala	1	15,201	51,177	1,262	828	449
Guinea	3	9,125	40,741	2,221	2,141	2,192
Guyana	1	2,216	6,994	180	109	29
Haiti	3	12,505	45,058	1,718	1,537	1,302
Honduras	2	27,740	95,071	2,155	1,403	989
India	1	80,319	243,975	12,512	5,648	4,299
Jordan	3	17,156	69,717	1,064	519	175
Kenya	3	20,311	78,278	2,583	2,430	1,667
Kyrgyzstan	1	5,531	16,004	334	256	52
Lesotho	3	5,849	17,175	781	513	251
Liberia	1	4,676	19,865	993	1,684	755
Madagascar	2	10,459	39,250	1,335	1,429	1,144
Malawi	3	19,118	74,200	3,329	4,180	3,221
Maldives	2	7,368	23,043	630	269	152
Mali	2	14,157	65,340	4,056	3,836	4,683
Moldova	1	4,745	9,560	175	106	49
Morocco	1	8,433	31,656	1,497	864	359
Mozambique	2	17,698	67,869	3,717	4,413	2,712
Myanmar	1	7,533	22,274	1,065	666	400
Namibia	2	5,312	18,093	477	466	247
Nepal	2	11,948	38,862	2,186	1,129	921
Niger	2	7,524	36,547	1,603	2,382	2,648
Nigeria	4	61,537	279,227	14,008	14,194	17,757
Pakistan	2	7,924	34,780	1,785	788	385
Peru	5	77,609	237,818	6,440	4,579	3,043
Rwanda	3	10,962	43,913	1,935	1,904	2,188
Senegal	2	6,120	25,867	1,174	927	1,168
Sierra Leone	1	2,540	9,648	541	742	402
South Africa	1	949	2,611	78	59	24
São Tomé and Príncipe	1	1,937	7,402	149	267	161
Tajikistan	1	6,126	19,784	481	585	175
Tanzania	3	21,525	88,717	3,340	3,660	2,872
Timor-Leste	1	7,820	35,370	1,472	1,408	801
Togo	1	3,277	12,769	486	383	538
Turkey	2	8,750	24,889	686	503	158
Uganda	2	3,785	17,914	751	996	746
Zambia	1	4,710	19,783	804	1,068	796
Zimbabwe	2	11,729	35,889	950	837	566
eSwatini	1	2,211	8,712	232	352	167
**Totals**						
**58**	**114**	**875,396**	**3,112,474**	**124,558**	**103,957**	**90,104**

Supplementary Table 2

Percent reduction in childhood mortality across age intervals by parent's gender (all effect sizes use a reference group of 0 years of parental education).

Independent Variable	Age Interval	6 Yrs. of Schooling	12 Yrs. of Schooling	16 Yrs. of Schooling
Father's Education	0 to 11 Months	8.24% (7.07% - 8.86%)	15.8% (13.6% - 16.9%)	20.5% (17.8% - 21.9%)
Father's Education	0 to 27 Days	6.38% (4.72% - 6.96%)	12.3% (9.22% - 13.4%)	16.1% (12.1% - 17.5%)
Father's Education	0 to 4 Years	9.08% (7.81% - 9.88%)	17.3% (15.0% - 18.8%)	22.4% (19.5% - 24.2%)
Father's Education	1 Month to 4 Years	11.4% (9.82% - 12.5%)	21.4% (18.7% - 23.5%)	27.5% (24.1% - 30.1%)
Father's Education	1 to 11 Months	10.3% (9.00% - 11.4%)	19.6% (17.2% - 21.4%)	25.3% (22.2% - 27.5%)
Father's Education	1 to 4 Years	12.4% (11.0% - 13.4%)	23.3% (20.7% - 25.0%)	29.8% (26.6% - 31.9%)
Mother's Education	0 to 11 Months	16.5% (15.2% - 17.6%)	30.3% (28.0% - 32.1%)	38.2% (35.5% - 40.3%)
Mother's Education	0 to 27 Days	8.59% (7.35% - 9.47%)	16.4% (14.2% - 18.0%)	21.3% (18.4% - 23.3%)
Mother's Education	0 to 4 Years	16.9% (15.8% - 17.9%)	31.0% (29.0% - 32.6%)	39.0% (36.7% - 40.9%)
Mother's Education	1 Month to 4 Years	20.9% (19.6% - 22.1%)	37.5% (35.3% - 39.4%)	46.5% (44.0% - 48.7%)
Mother's Education	1 to 11 Months	20.2% (18.5% - 21.4%)	36.3% (33.5% - 38.2%)	45.2% (42.0% - 47.3%)
Mother's Education	1 to 4 Years	23.5% (22.3% - 25.7%)	41.5% (39.7% - 44.8%)	51.0% (49.0% - 54.7%)

Supplementary Figure 1

Normalized input data, superimposed with estimated average effect sizes

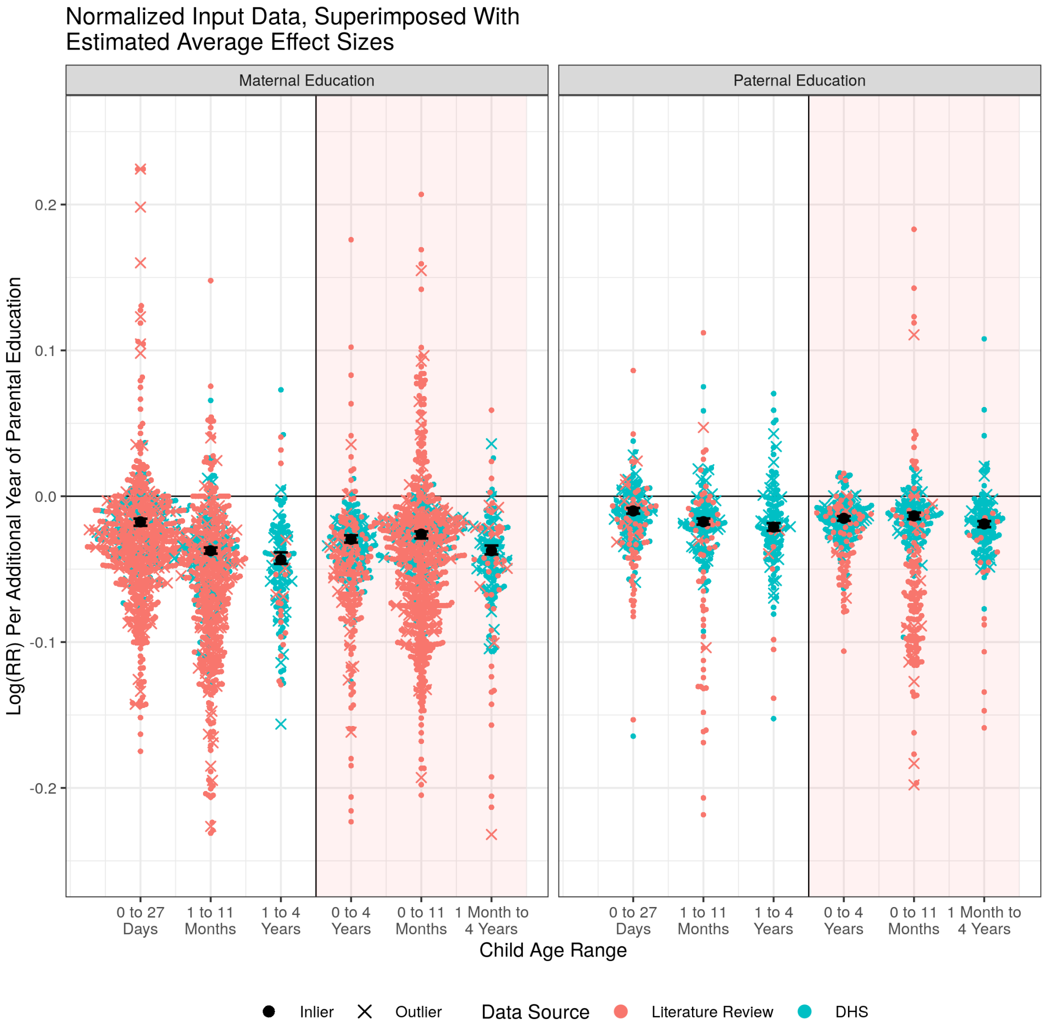

Supplementary Figure 2

Relative risk of under-5 mortality, by age group and parents' education

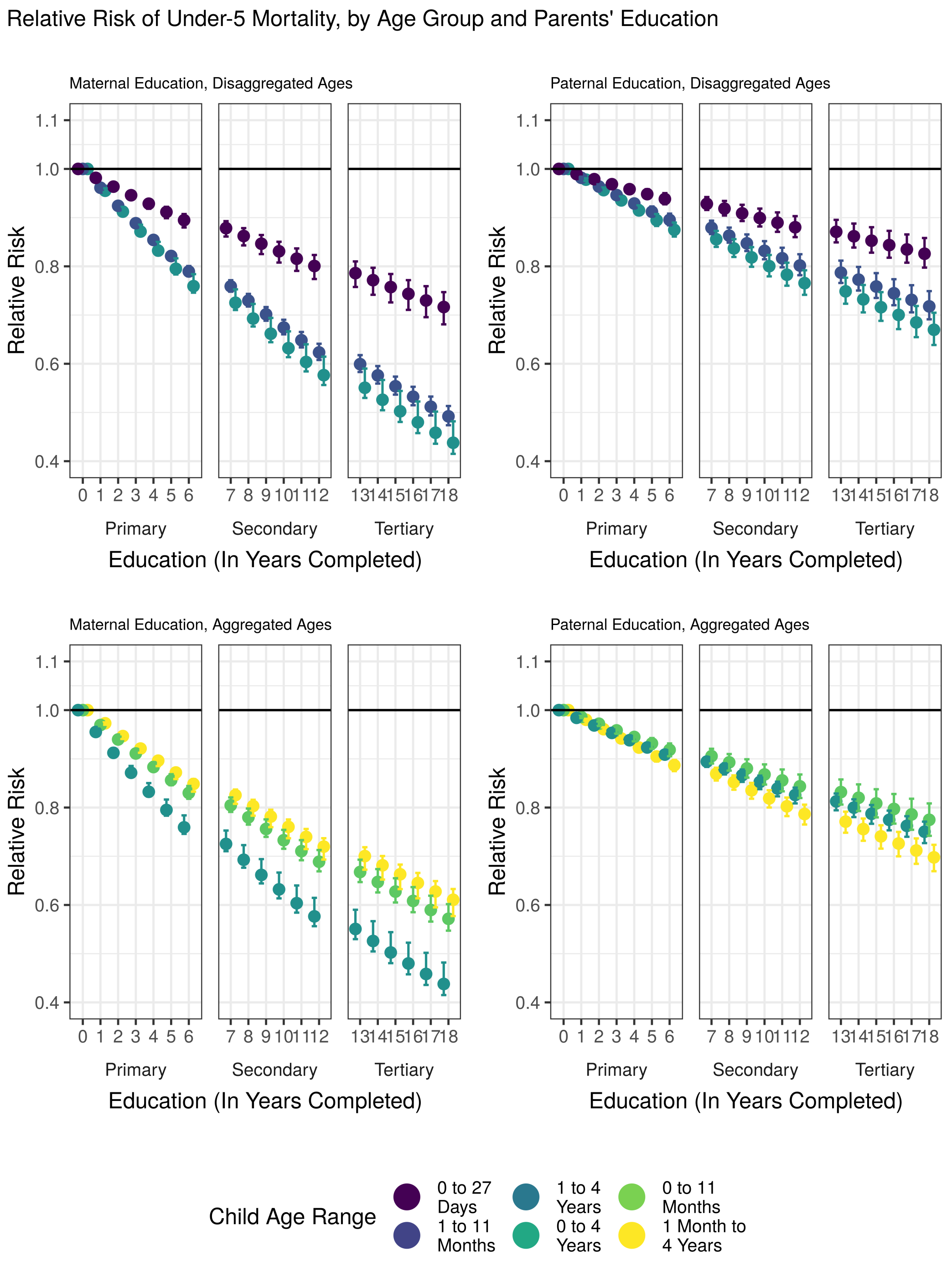

Supplementary Figure 3

Normalized relative risks per one year of parental education, by age group, parent's education, and data source.

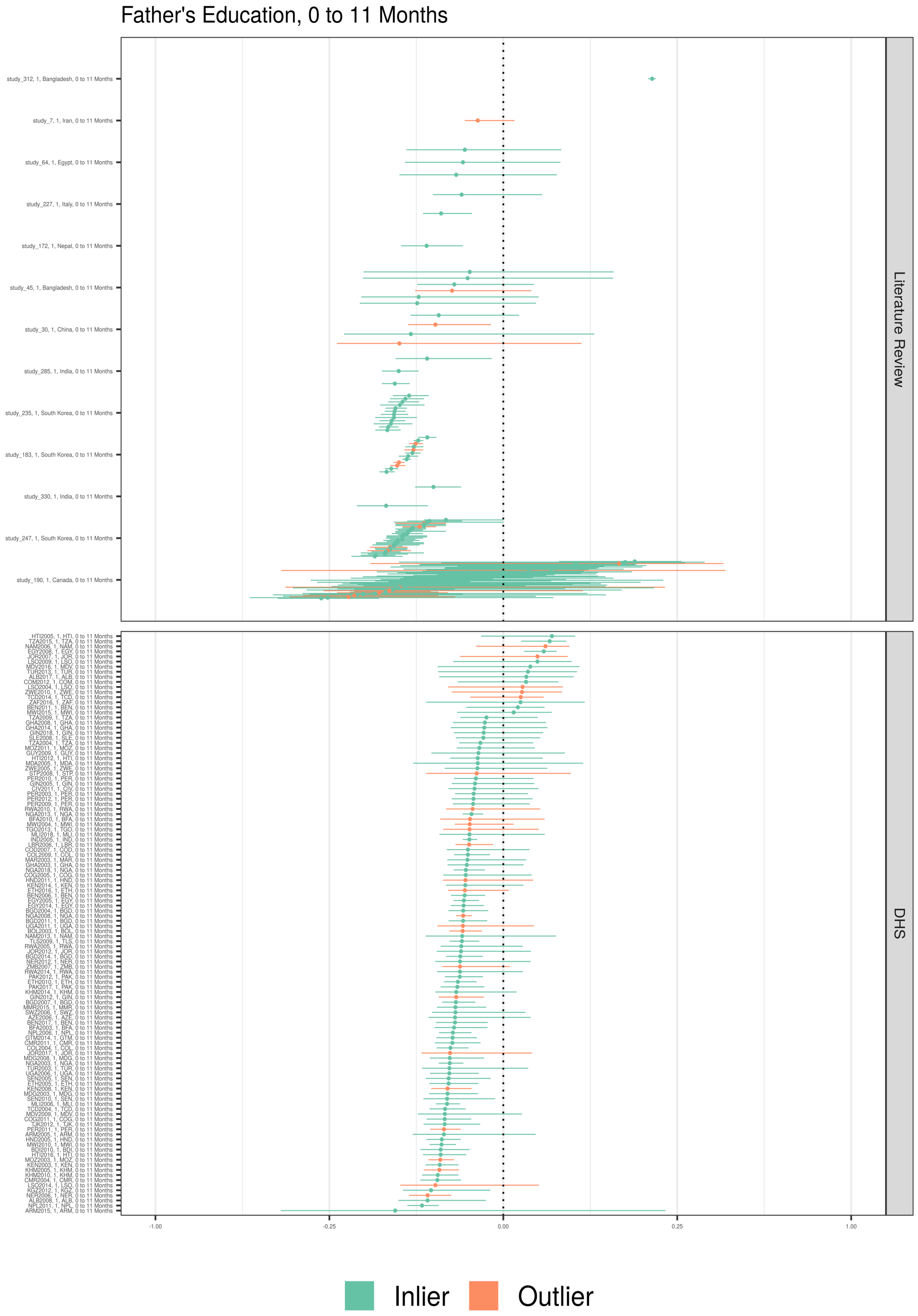

Supplementary Figure 4

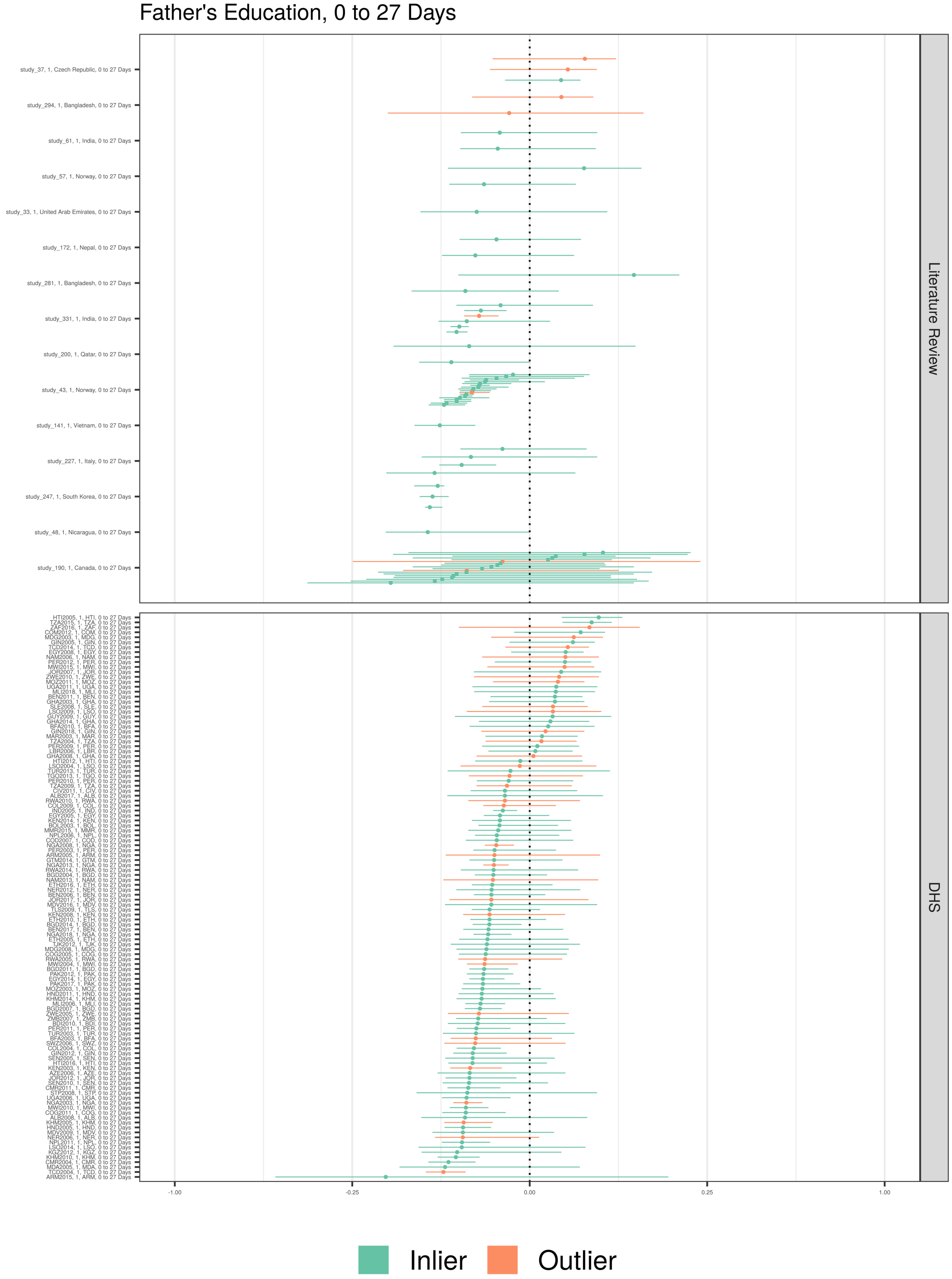

Supplementary Figure 5

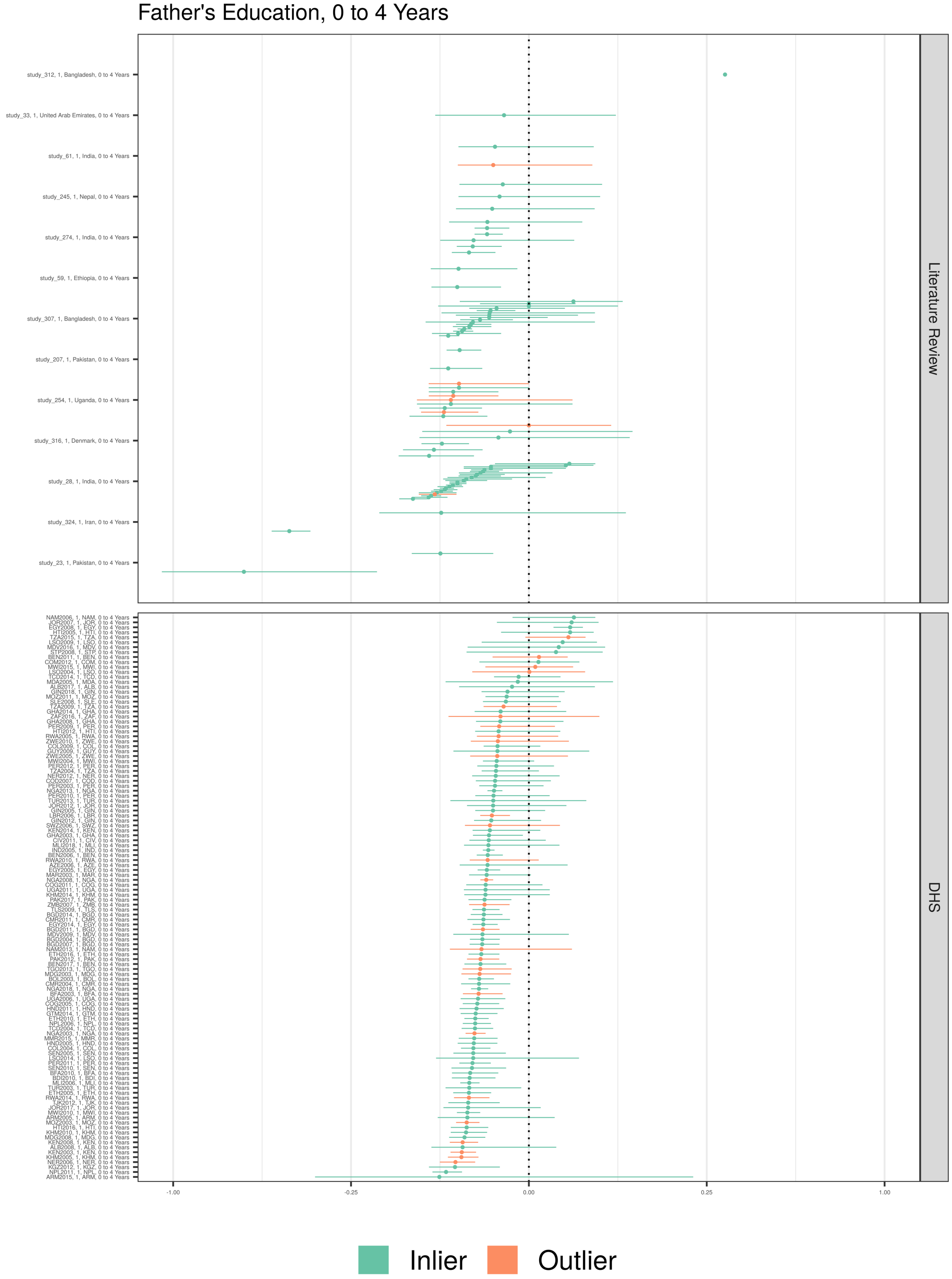

Supplementary Figure 6

Father's Education, 1 Month to 4 Years

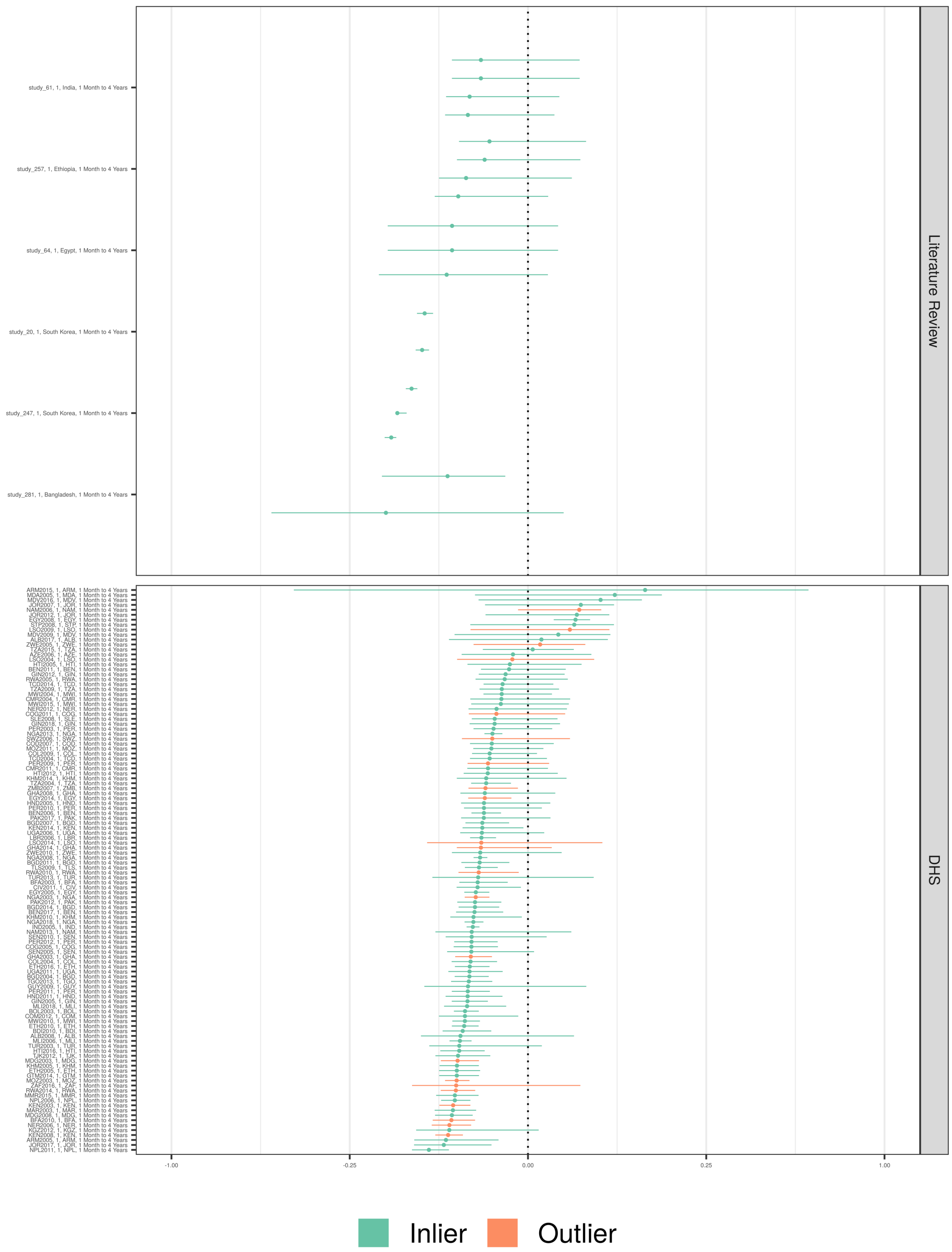

Supplementary Figure 7

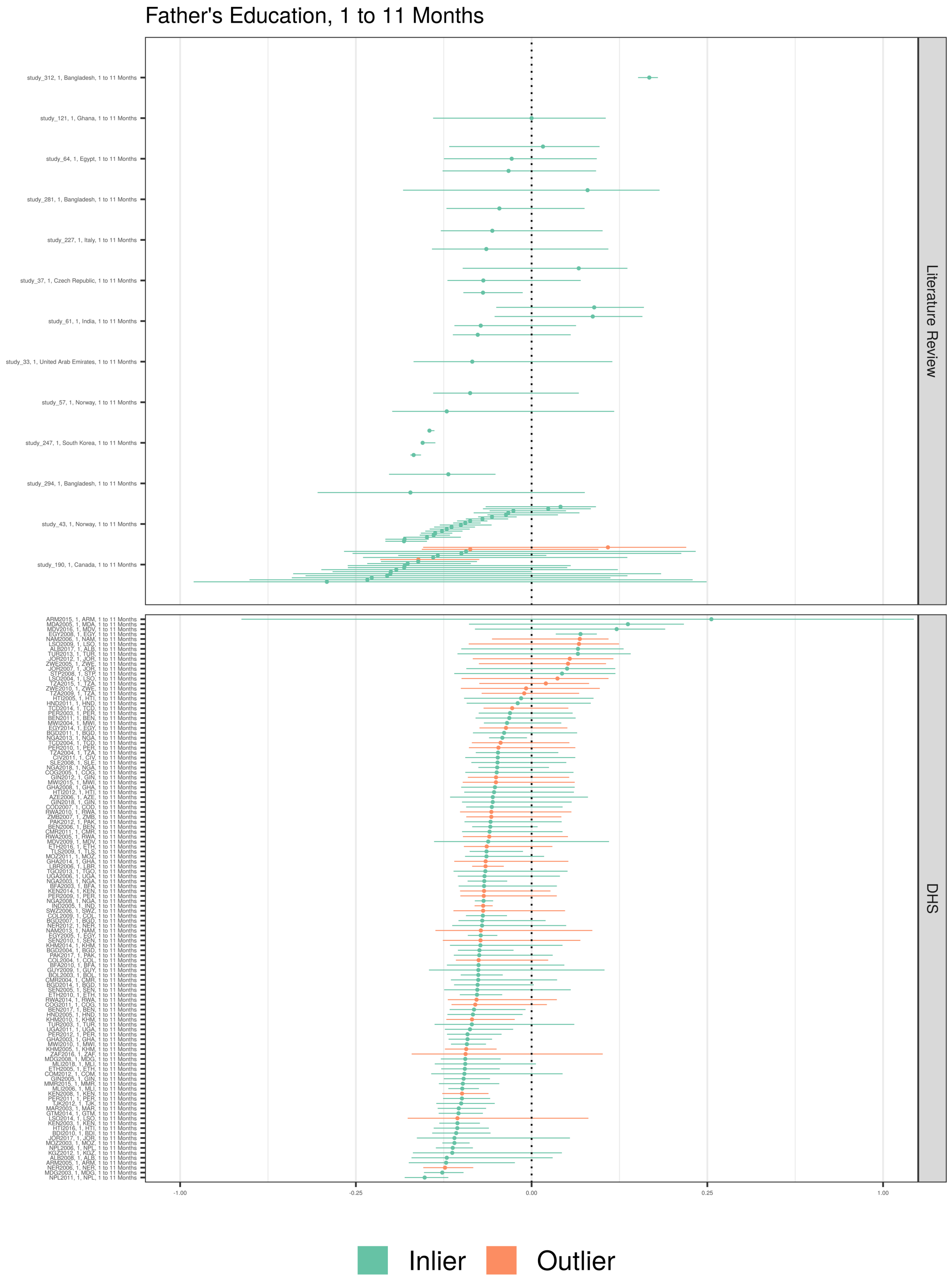

Supplementary Figure 8

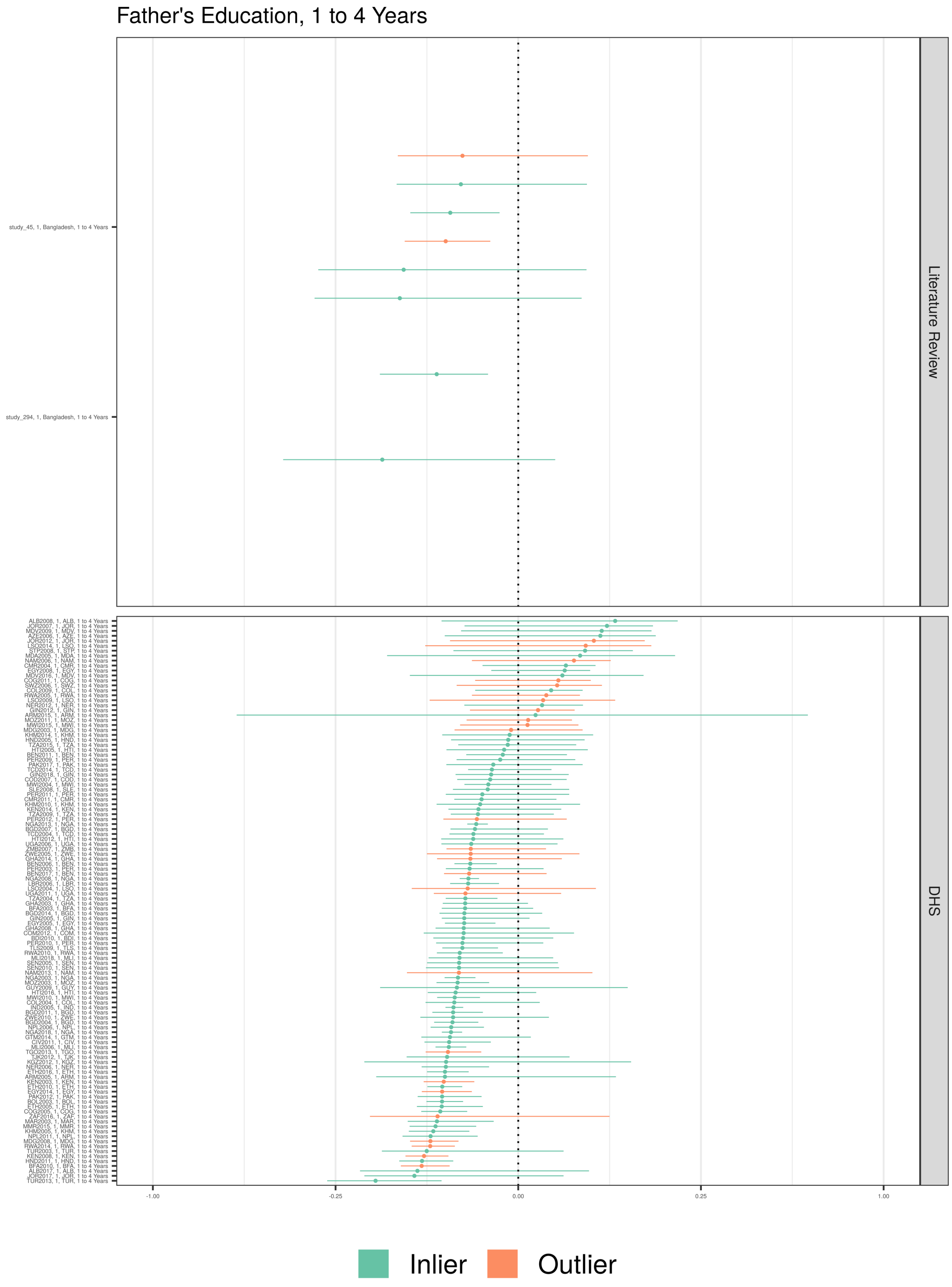

Supplementary Figure 9

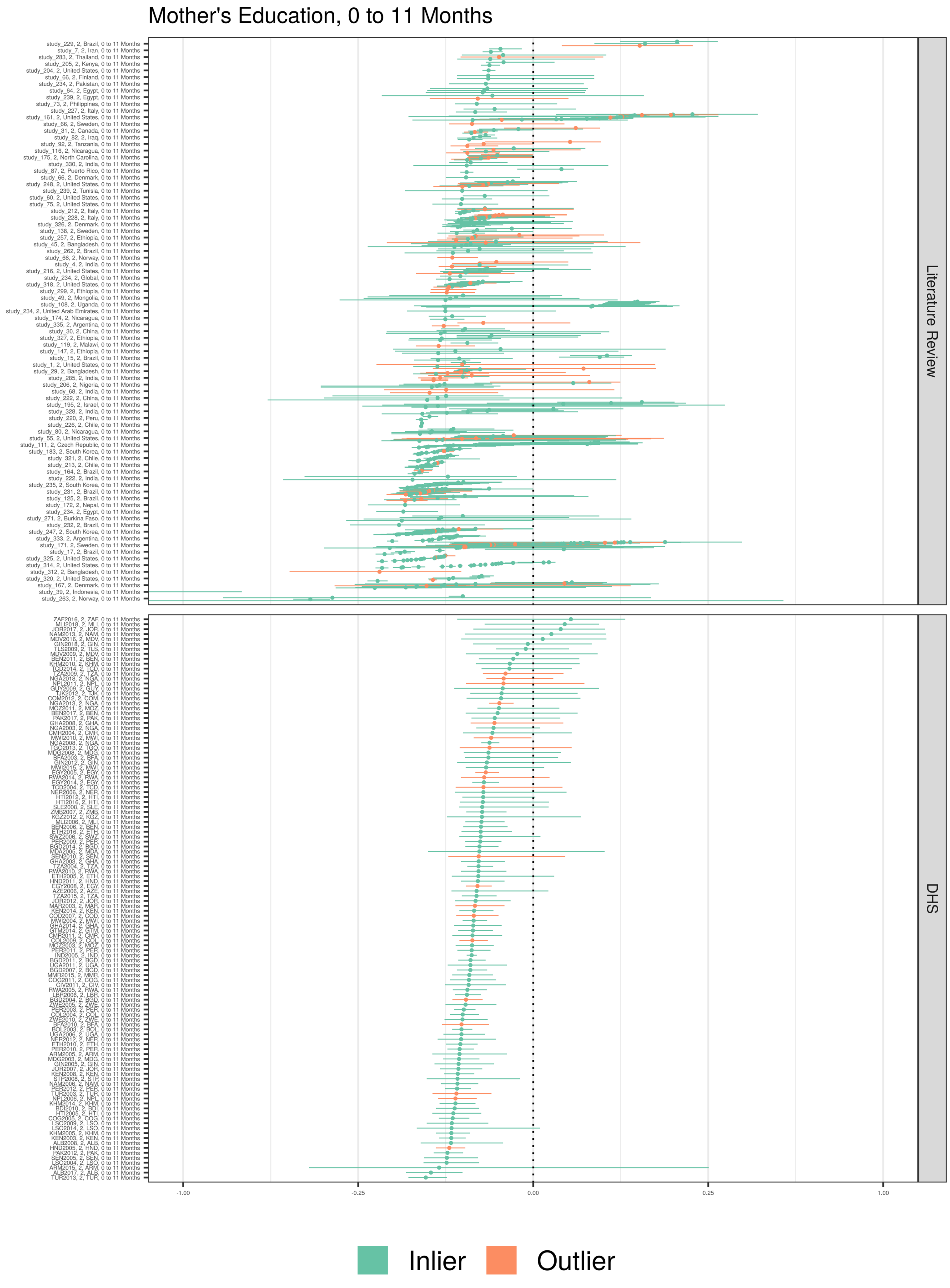

Supplementary Figure 10

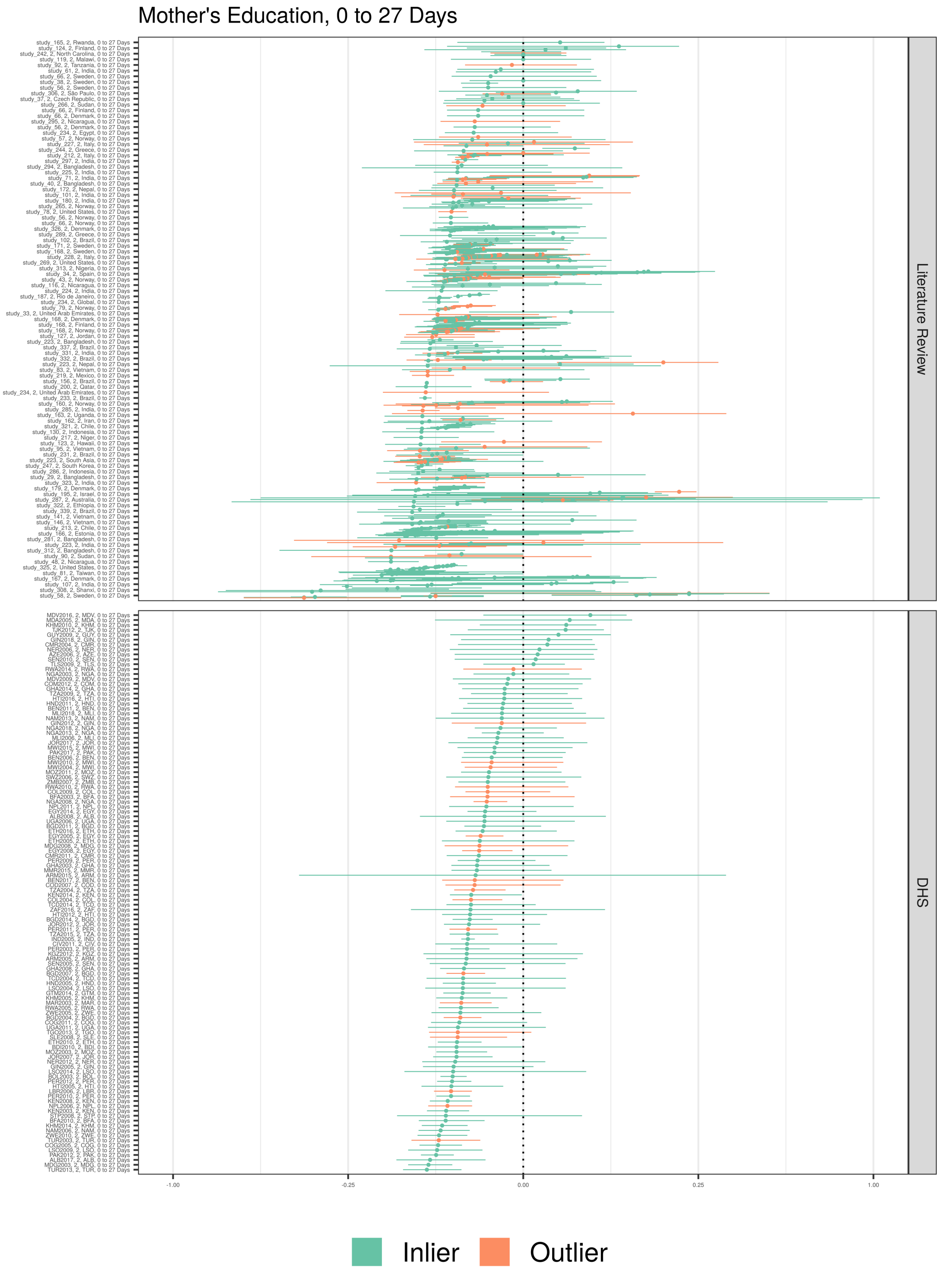

Supplementary Figure 11

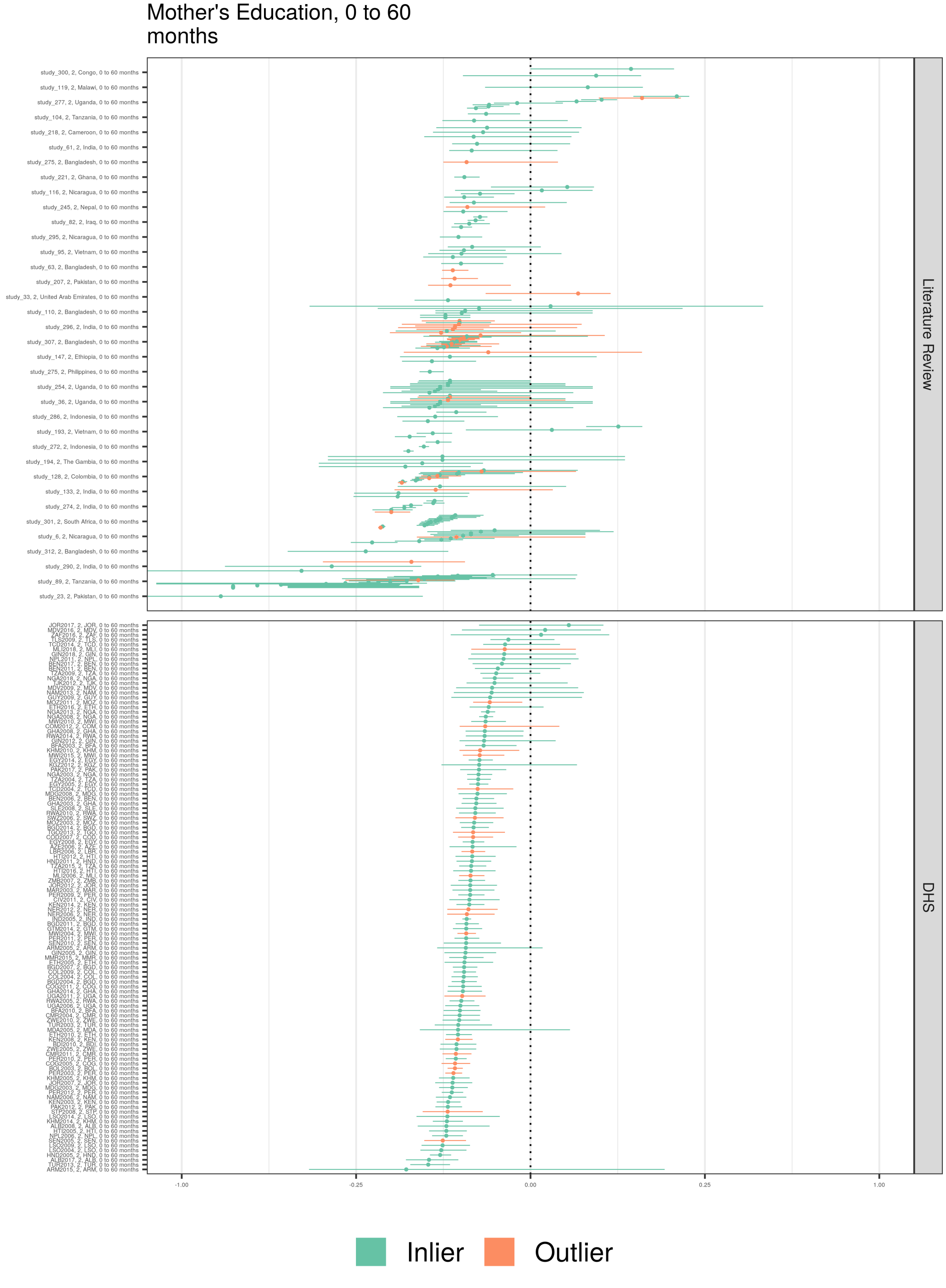

Supplementary Figure 12

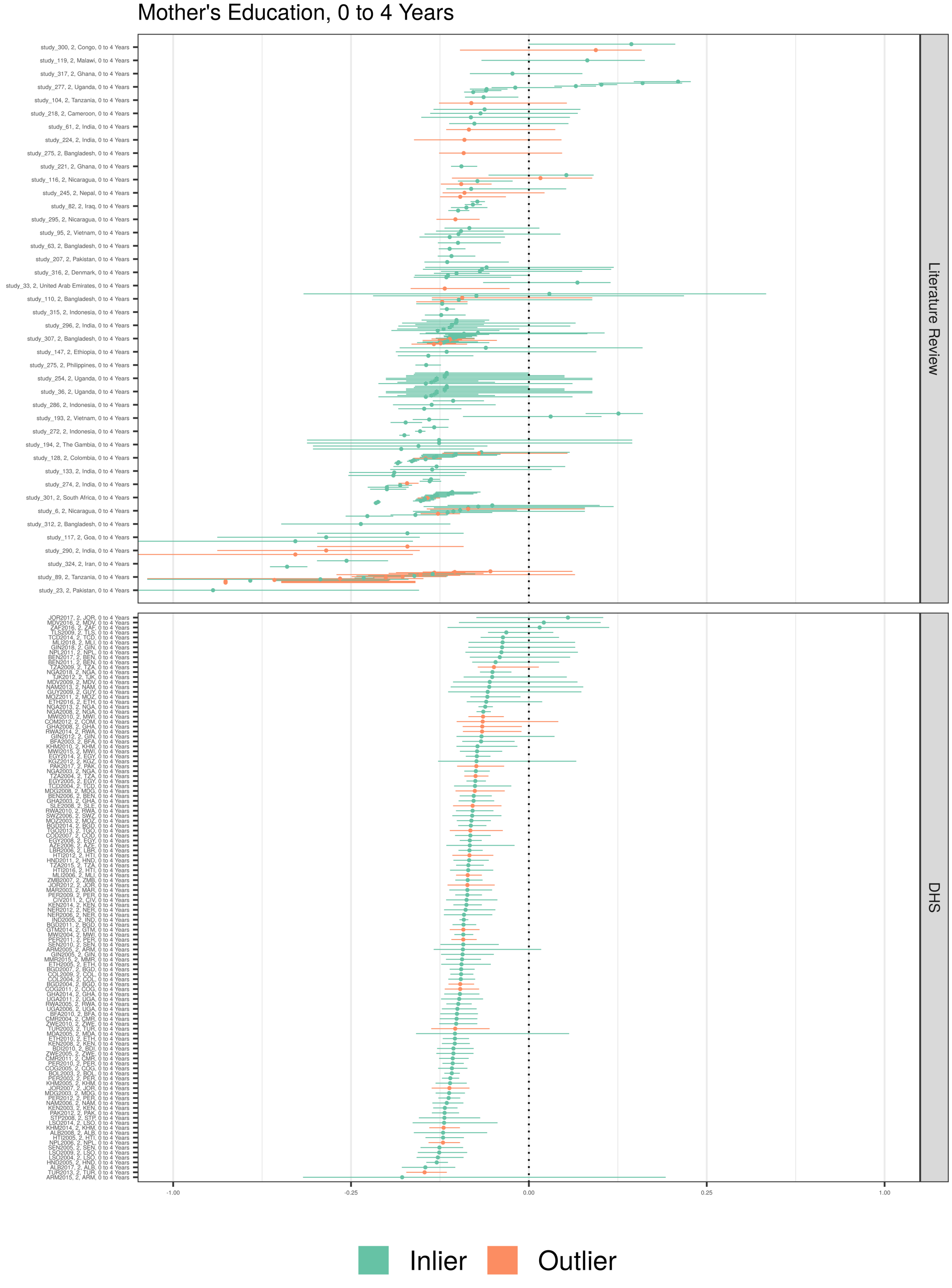

Supplementary Figure 13

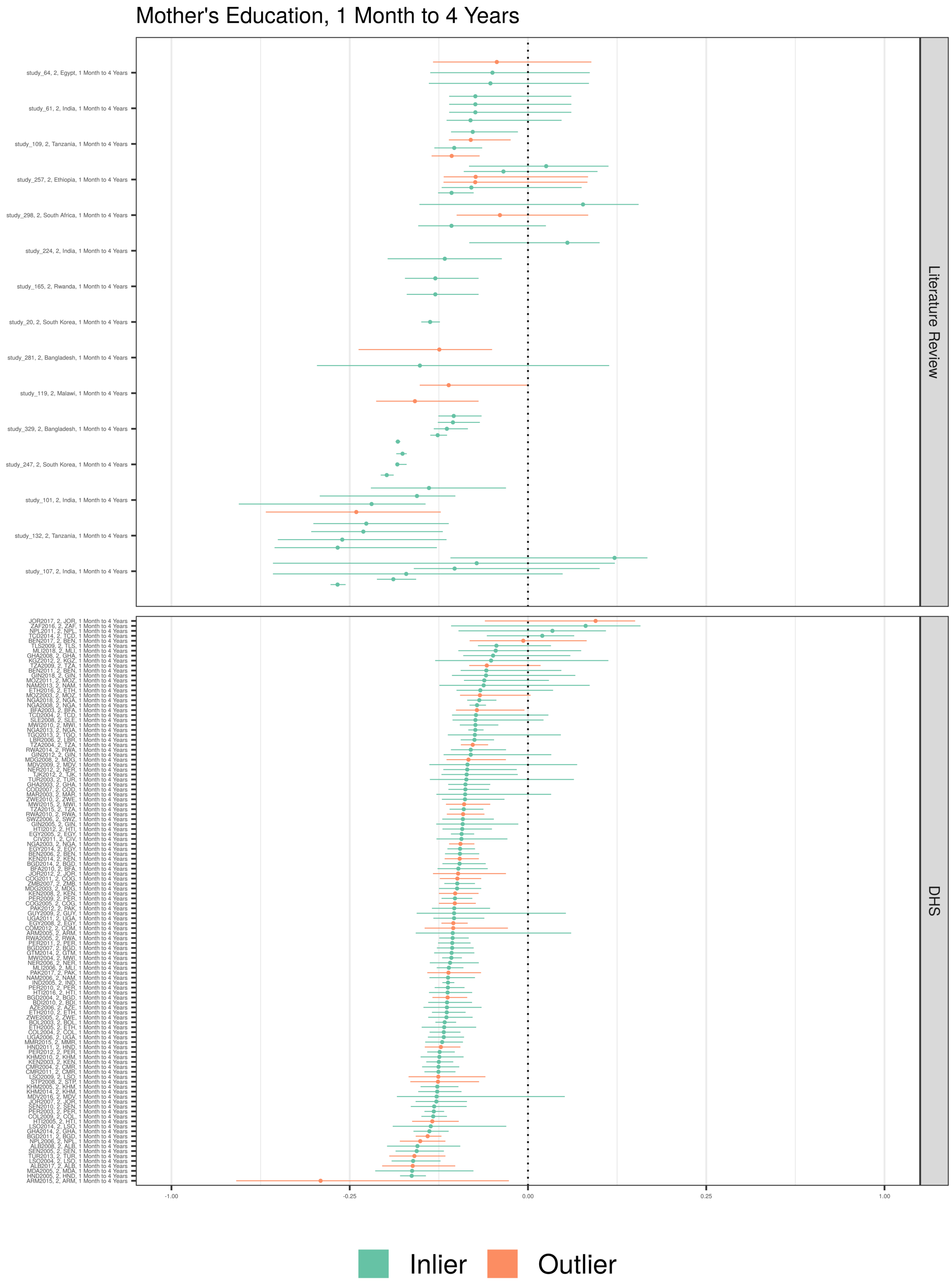

Supplementary Figure 14

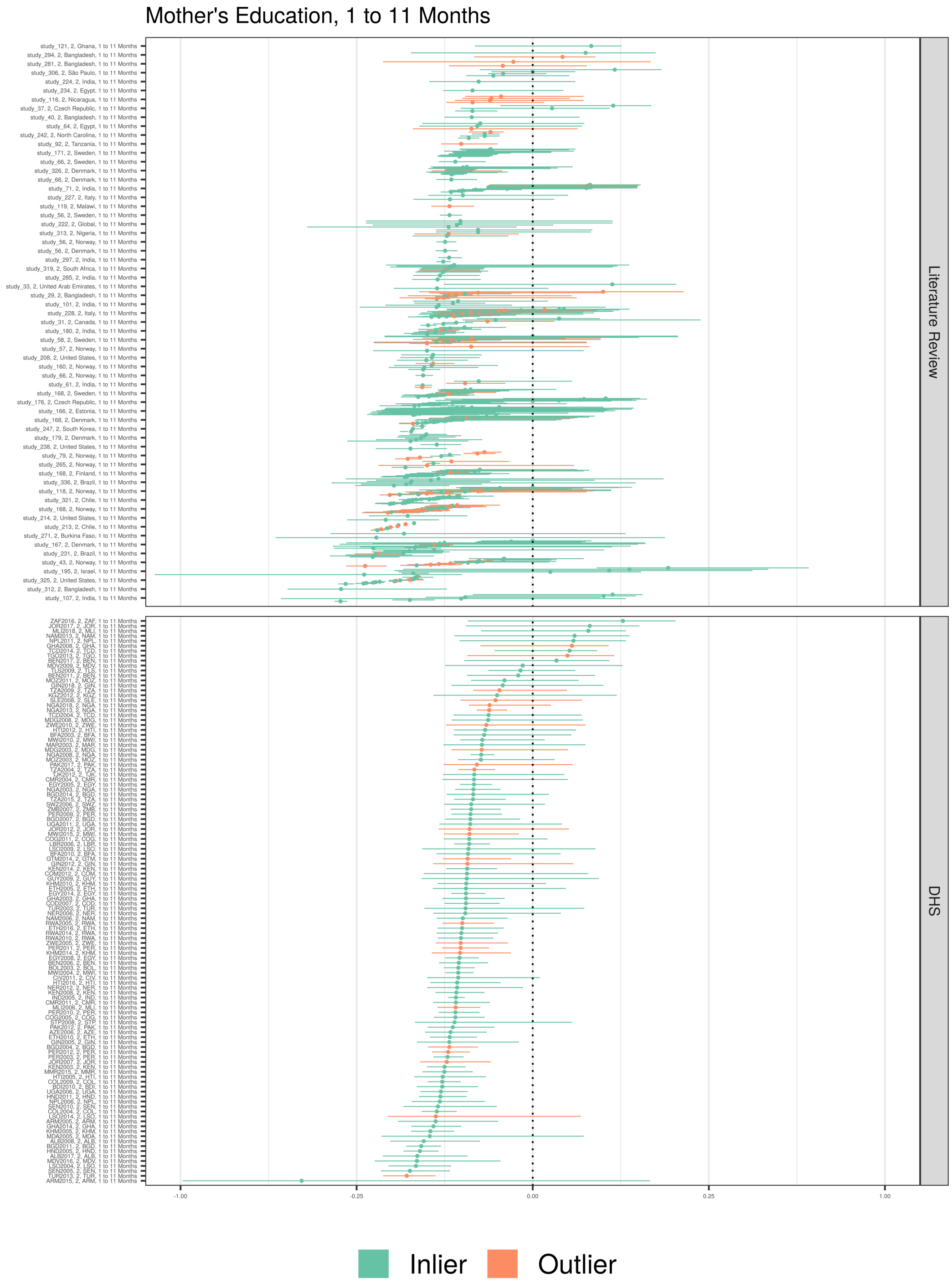

Supplementary Figure 15

Sensitivity analysis of DHS Microdata Primary Analyses

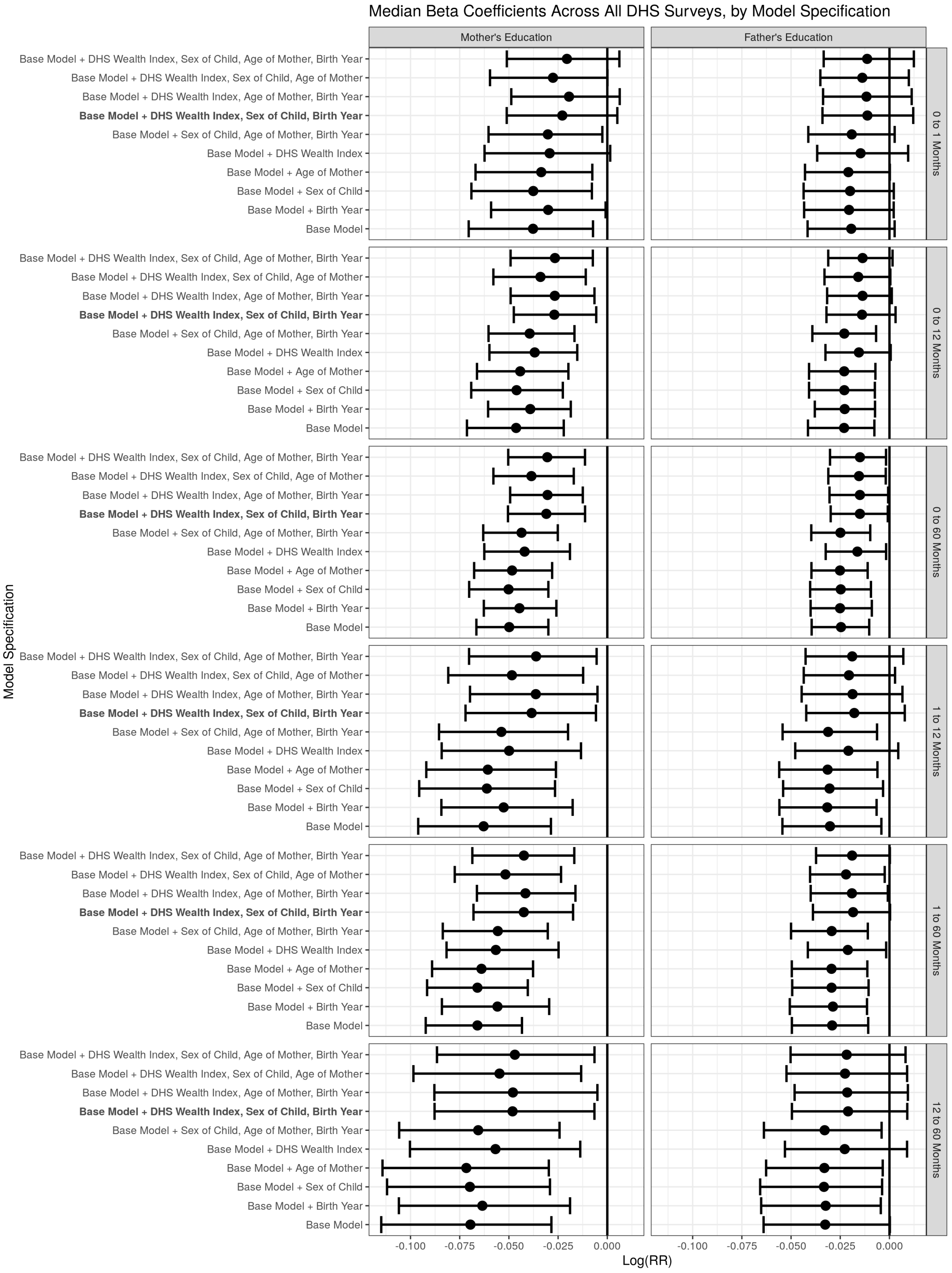

Supplementary Figure 16

Sensitivity analysis of Systematic Review + DHS Synthesis and Meta-Analysis

'Log(RR) Per Additional Year of Parental Education' is identical to the beta value estimated by the model.

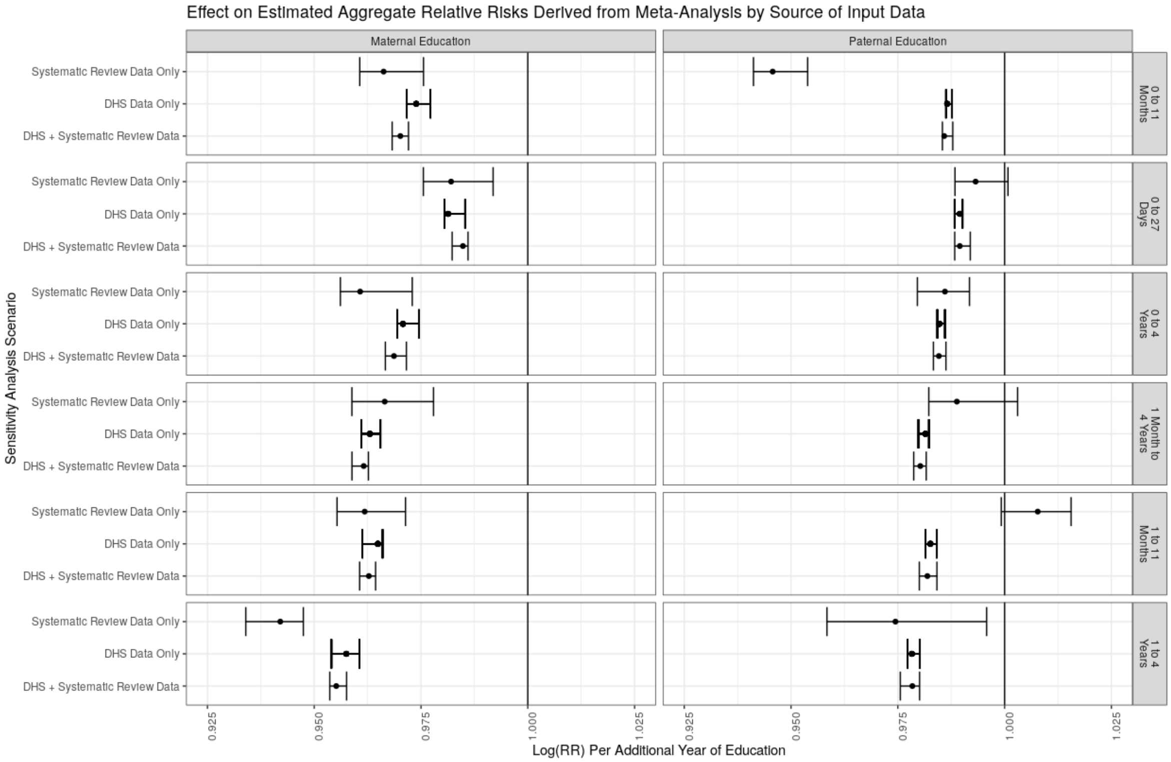

8. List of articles included in systematic review

1) Adetunji JA. Infant mortality in Nigeria: effects of place of birth, mother's education and region of residence. Journal of Biosocial Science. 1994;26(4):469-77.
2) Adeyinka DA, Muhajarine N, Petrucka P, Isaac EW. Inequities in child survival in Nigerian communities during the Sustainable Development Goal era: insights from analysis of 2016/2017 Multiple Indicator Cluster Survey. BMC Public Health. 2020 Oct 27;20(1):1613.
3) Adlakha AL, Suchindran CM. Factors affecting infant and child mortality. Journal of Biosocial Science. 1985;17(4):481-96.
4) Agha A, Ajmal F, Iqbal A, White F. Father's support and literacy--factors associated with child mortality in Gambat, Sindh-Pakistan. Journal of the Pakistan Medical Association. 2010;60(2):81.
5) Aguilera X, Delgado I, Icaza G, Apablaza M, Villanueva L, Castillo-Laborde C. Under five and infant mortality in Chile (1990-2016): Trends, disparities, and causes of death. PLoS One. 2020 Sep 30;15(9):e0239974.
6) Akter T, Hoque DME, Chowdhury EK, Rahman M, Russell M, Arifeen S. Is there any association between parental education and child mortality? A study in a rural area of Bangladesh. Public health. 2015;129(12):1602-9.
7) Al Hosani, H.A., Brebner, J., Bener, A.B. & Norman, J.N. Study of mortality risk factors for children under age 5 in Abu Dhabi. Eastern Mediterranean health journal. 2003;9(3):333.
8) Alam N, Van Ginneken JK, Bosch AM. Infant mortality among twins and triplets in rural Bangladesh in 1975–2002. Tropical Medicine & International Health. 2007;12(12):1506-14.
9) Alam N. Teenage motherhood and infant mortality in Bangladesh: maternal age-dependent effect of parity one. Journal of biosocial science. 2000;32(2):229-36.
10) Aleman J, Liljestrand J, Peña R, Wall S, Persson L. Which Babies Die during the First Week? Gynecologic and obstetric investigation. 1997;43(2):112-5.
11) Alexander GR, Baruffi G, Mor JM, Kieffer E. Pregnancy outcomes among Whites and Filipinos: A paradoxical birth weight-neonatal mortality relationship. Am J Hum Biol. 1993;5(2):203-209.
12) Ali MM, Shah IH. Sanctions and childhood mortality in Iraq. The lancet. 2000;355(9218):1851-7.
13) Amin S. The effect of women's status on sex differentials in infant and child mortality in South Asia. Genus. 1990 Jul-Dec;46(3-4):55-69.
14) Andrade CLTd, Szwarcwald CL, Gama SGNd, Leal MdC. Socioeconomic inequalities and low birth weight and perinatal mortality in Rio de Janeiro, Brazil. Cadernos de Saúde Pública. 2004;20:S44-S51.
15) Armstrong Schellenberg JR, Nathan R, Abdulla S, Mukasa O, Marchant TJ, Tanner M, et al. Risk factors for child mortality in rural Tanzania. Tropical Medicine & International Health. 2002;7(6):506-11.

16) Arntzen A, Magnus P, Bakketeig LS. Different effects of maternal and paternal education on early mortality in Norway. Paediatric and perinatal epidemiology. 1993;7(4):376-86.
17) Arntzen A, Mortensen L, Schnor O, Cnattingius S, Gissler M, Andersen A-MN. Neonatal and postneonatal mortality by maternal education—a population-based study of trends in the Nordic countries, 1981–2000. European Journal of Public Health. 2008;18(3):245-51.
18) Arntzen A, Moum T, Magnus P, Bakketeig L. The association between maternal education and postneonatal mortality. Trends in Norway, 1968–1991. International journal of epidemiology. 1996;25(3):578-84.
19) Arntzen A, Moum T, Magnus P, Bakketeig LS. Marital status as a risk factor for fetal and infant mortality. Scandinavian journal of social medicine. 1996;24(1):36-42.
20) Arntzen A, Samuelsen SO, Bakketeig LS, Stoltenberg C. Parents' education and infant mortality 1967-1998. Tidsskrift for den Norske laegeforening: tidsskrift for praktisk medicin, ny raekke. 2004;124(22):2904-6.
21) Arntzen A, Samuelsen SO, Bakketeig LS, Stoltenberg C. Socioeconomic status and risk of infant death. A population-based study of trends in Norway, 1967–1998. International Journal of Epidemiology. 2004;33(2):279-88.
22) Asefa M, Drewett R, Tessema F. A birth cohort study in South-West Ethiopia to identify factors associated with infant mortality that are amenable for intervention. Ethiopian Journal of Health Development, 2000, 14(2): 161-168
23) Bakketeig LS, Cnattingius S, Knudsen LB. Socioeconomic differences in fetal and infant mortality in Scandinavia. Journal of public health policy. 1993;14(1):82-90.
24) Bashir AO, Ibrahim GH, Bashier IA, Adam I. Neonatal mortality in Sudan: analysis of the Sudan household survey, 2010. BMC Public Health. 2013;13(1):287.
25) Basu AM, Stephenson R. Low levels of maternal education and the proximate determinants of childhood mortality: a little learning is not a dangerous thing. Social science & medicine. 2005;60(9):2011-23.
26) Bawah AA, Awoonor-Williams JK, Asuming PO, Jackson EF, Boyer CB, Kanmiki EW, Achana SF, Akazili J, Phillips JF. The child survival impact of the Ghana Essential Health Interventions Program: A health systems strengthening plausibility trial in Northern Ghana. PLoS One. 2019 Jun 12;14(6):e0218025.
27) Bayasgalan B. Determinants of infant and child mortality in Mongolia. Mong J Demogr. 1996 Oct;1(1):12-26.
28) Becerra JE, Atrash HK, Pérez N, Saliceti JA. Low birthweight and infant mortality in Puerto Rico. American journal of public health. 1993;83(11):1572-6.
29) Sosnaud B. Inequality in Infant Mortality: Cross-State Variation and Medical System Institutions, Social Problems, Volume 66, Issue 1, February 2019, 108–127.
30) Bennett J. Correlates of child mortality in Pakistan: a hazards model analysis. The Pakistan Development Review. 1999:85-118.
31) Bhuiya A, Streatfield K. A hazard logit model analysis of covariates of childhood mortality in Matlab, Bangladesh. Journal of biosocial science. 1992;24(4):447-62.
32) Bilsteen JF, Andresen JB, Mortensen LH, Hansen AV, Andersen A-MN. Educational disparities in perinatal health in Denmark in the first decade of the 21st century: a register-based cohort study. BMJ open. 2018;8(11).

33) Binka FN, Maude GH, Gyapong M, Ross DA, Smith PG. Risk factors for child mortality in northern Ghana: a case-control study. International Journal of Epidemiology. 1995;24(1):127-35.
34) Bloland P, Slutsker L, Steketee RW, Wirima JJ, Heymann DL, Breman JG. Rates and risk factors for mortality during the first two years of life in rural Malawi. The American journal of tropical medicine and hygiene. 1996;55(1_Suppl):82-6.
35) Borrell C, Cirera E, Ricart M, Pasarín M, Salvador J. Social inequalities in perinatal mortality in a Southern European city. European journal of epidemiology. 2003;18(1):5-13.
36) Brahmbhatt H, Bishai D, Wabwire-Mangen F, Kigozi G, Wawer M, Gray RH, et al. Polygyny, maternal HIV status and child survival: Rakai, Uganda. Social science & medicine. 2002;55(4):585-92.
37) Broeck Jvd, Eeckels R, Massa G. Maternal determinants of child survival in a rural African community. International journal of epidemiology. 1996;25(5):998-1004.
38) Bross DS, Shapiro S. Direct and indirect associations of five factors with infant mortality. American Journal of Epidemiology. 1982;115(1):78-91.
39) Buwembo P. Factors associated with under-5 mortality in South Africa: Trends 1997-2002: University of Pretoria; 2010.
40) Calling S, Li X, Sundquist J, Sundquist K. Socioeconomic inequalities and infant mortality of 46 470 preterm infants born in Sweden between 1992 and 2006. Paediatric and perinatal epidemiology. 2011;25(4):357-65.
41) Carlsen F, Grytten J, Eskild A. Maternal education and risk of offspring death; changing patterns from 16 weeks of gestation until one year after birth. The European Journal of Public Health. 2014;24(1):157-62.
42) Casterline JB, Cooksey EC, Ismail AF. Infant and child mortality in rural Egypt. Journal of biosocial science. 1992;24(2):245-60.
43) Chen J, Xie Z, Liu H. Son preference, use of maternal health care, and infant mortality in rural China, 1989–2000. Population studies. 2007;61(2):161-83.
44) Choe MK, Retherford RD, Thapa S, Gubhaju BB. Ethnic differentials in early childhood mortality in Nepal. 1987.
45) Chowdhury QH, Islam R, Hossain K. Socio-economic determinants of neonatal, post neonatal, infant and child mortality. International Journal of Sociology and Anthropology. 2010;2(6):118-25.
46) Cleland J, van Ginneken J. Maternal schooling and childhood mortality. Journal of Biosocial Science. 1989;21(S10):13-34.
47) Cnattingius S, Haglund B. Socio-economic factors and feto-infant mortality. Scandinavian journal of social medicine. 1992;20(1):11-3.
48) Damghanian M, Shariati M, Mirzaiinajmabadi K, Yunesian M, Emamian MH. Socioeconomic inequality and its determinants regarding infant mortality in Iran. Iranian Red Crescent Medical Journal. 2014;16(6).
49) Dashtseren A, editor Determinants of infant and child mortality in Mongolia. IUSSP Regional Conference, Bangkong, Thailand; 2002.
50) Deb AK, Dutta S, Hnichho C, Vanlalpeki M, Phosa HT, Rakhu K, et al. A case control study investigating factors associated with high infant death in Saiha district of Mizoram, India bordering Myanmar. BMC pediatrics. 2017;17(1):23.

51) Diallo AH, Meda N, Sommerfelt H, Traore GS, Cousens S, Tylleskar T, et al. The high burden of infant deaths in rural Burkina Faso: a prospective community-based cohort study. BMC Public Health. 2012;12(1):739.
52) Din-Dzietham R, Hertz-Picciotto I. Infant mortality differences between whites and African Americans: the effect of maternal education. Am J Public Health. 1998 Apr;88(4):651-6.
53) Din-Dzietham R, Hertz-Picciotto I. Relationship of education to the racial gap in neonatal and postneonatal mortality. Arch Pediatr Adolesc Med. 1997 Aug;151(8):787-92.
54) D'Souza, S., & Bhuiya, A. (1982). Socioeconomic Mortality Differentials in a Rural Area of Bangladesh. Population and Development Review, 8(4), 753-769.
55) El Ansari W, Su R, Nimeri N, Latiph E, Yousafzai MT, Tohid H. Level of maternal education is a significant determinant of neonatal survival: A PEARL study analysis. Journal of the College of Physicians and Surgeons--Pakistan: JCPSP. 2015;25(2):151-3.
56) Emuren L, Chauhan S, Vroman R, Beydoun H. Epidemiology of infant death among black and white non-Hispanic populations in Hampton Roads, Virginia. Southern medical journal. 2012;105(5):259-65.
57) Fonseca SC, Flores PVG, Camargo KR Jr, Pinheiro RS, Coeli CM. Maternal education and age: inequalities in neonatal death. Rev Saude Publica. 2017 Nov 17;51:94.
58) Frenz P, González C. Application of a simple methodological approach to analyze health inequalities: the case of infant mortality in Chile. Revista medica de Chile. 2010;138(9):1157-64.
59) Frenzen PD, Hogan DP. The impact of class, education, and health care on infant mortality in a developing society: the case of rural Thailand. Demography. 1982;19(3):391-408.
60) Gage TB, Fang F, O'Neill E, DiRienzo G. Maternal education, birth weight, and infant mortality in the United States. Demography. 2013;50(2):615-35.
61) Garba M, Kamaye M, Alido S, Zoubeirou H, Oumarou Z, Amadou A. Determinants of early neonatal mortality in Issaka-Gazobi maternity of Niamey. Journal de Pediatrie et de Puericulture. 2017;30:156-61.
62) Ghaedmohammadi Z, Anaraki A, Khajeian A, Khajehian M, Ostovar A. Association of caesarean section and neonatal death: a population-based case-control study in Islamic Republic of Iran. EMHJ-Eastern Mediterranean Health Journal. 2015;21(4):266-72.
63) Gisselmann MD. Education, infant mortality, and low birth weight in Sweden 1973—1990: Emergence of the low birth weight paradox. Scandinavian journal of public health. 2005;33(1):65-71.
64) Gnavi R, Costa G. Pregnancy outcome, infant mortality and mother's education in Piedmont from 1980 to 1995. Epidemiologia e prevenzione. 2002;26(5):225-33.
65) Goldberg H, Rodrigues W, Thome A, Janowitz B, Morris L. Infant mortality and breast-feeding in north-eastern Brazil. Population Studies. 1984;38(1):105-15.
66) Greenspan A. Changes in fertility patterns can improve child survival in Southeast Asia. 1993.
67) Gruebner O, Lautenbach S, Khan M, Kipruto S, Epprecht M, Galea S. Place of residence moderates the risk of Infant Death in Kenya: Evidence from the most Recent Census 2009. PloS one. 2015;10(10):e0139545.

68) Guillot M, Allendorf K. Hindu-Muslim differentials in child mortality in India. Genus. 2010;66(2).
69) Gupta N, Hirschhorn LR, Rwabukwisi FC, Drobac P, Sayinzoga F, Mugeni C, et al. Causes of death and predictors of childhood mortality in Rwanda: a matched case-control study using verbal social autopsy. BMC public health. 2018;18(1):1378.
70) Haglund B, Cnattingius S, Nordström ML. Social differences in late fetal death and infant mortality in Sweden 1985–86. Paediatric and perinatal epidemiology. 1993;7(1):33-44.
71) Haidar FH, Oliveira UF, Nascimento L. Maternal educational level: correlation with obstetric indicators. Cadernos de saude publica. 2001;17(4):1025.
72) Hailemariam A, Tesfaye M. Determinants of infant and early childhood mortality in a small urban community of Ethiopia: a hazard model analysis. The Ethiopian Journal of Health Development (EJHD). 1997;11(3).
73) Helena, Ernani Tiaraju de Santa, Sousa, Clóvis Arlindo de, & Silva, Cristiane Amorin da. (2005). Fatores de risco para mortalidade neonatal em Blumenau, Santa Catarina: linkage entre bancos de dados. Revista Brasileira de Saúde Materno Infantil, 5(2), 209-217.
74) HEMMINKI E, MERILÄINEN J, MALIN M, RAHKONEN O, TEPERI J. Mother's education and perinatal problems in Finland. International journal of epidemiology. 1992;21(4):720-4.
75) Hoa DP, Nga NT, Målqvist M, Persson LÅ. Persistent neonatal mortality despite improved under-five survival: a retrospective cohort study in northern Vietnam. Acta paediatrica. 2008;97(2):166-70.
76) Holland MA, Young ML, Jiroutek MR. Racial and ethnic disparities in infant mortality in North Carolina, 2008–2009. North Carolina medical journal. 2016;77(6):373-7.
77) Hollstein RD, Vega J, Carvajal Y. [Social inequalities and health. Socioeconomic level and infant mortality in Chile in 1985-1995]. Rev Med Chil. 1998;126(3):333-40.
78) Hossain MB, Phillips JF, Pence B. The effect of women's status on infant and child mortality in four rural areas of Bangladesh. Journal of biosocial science. 2007;39(3):355.
79) Houweling TA, van Klaveren D, Das S, Azad K, Tripathy P, Manandhar D, et al. A prediction model for neonatal mortality in low-and middle-income countries: an analysis of data from population surveillance sites in India, Nepal and Bangladesh. International journal of epidemiology. 2019;48(1):186-98.
80) Howell EM, Vert P. Neonatal intensive care and birth weight-specific perinatal mortality in Michigan and Lorraine. Pediatrics. 1993;91(2):464-9.
81) Ibrahim S, Babiker AG, Amin I, Omer M, Rushwan H. Factors associated with high risk of perinatal and neonatal mortality: an interim report on a prospective community-based study in rural Sudan. Paediatric and perinatal Epidemiology. 1994;8(2):193-204.
82) Ifada L, Nurmalasari M, Pramana S. Multilevel survival analysis for under-fives in Indonesia 2015. PI. 24Apr.2020; 60(2):103
83) Jahan S. Poverty and infant mortality in the Eastern Mediterranean region: a meta-analysis. Journal of Epidemiology & Community Health. 2008;62(8):745-51.
84) Jensen A, Andersen PK, Andersen JS, Greisen G, Stensballe LG. Risk factors of post-discharge under-five mortality among Danish children 1997-2016: A register-based study. PLoS One. 2019 Dec 4;14(12):e0226045.

85) Jung-Choi K, Khang Y-H. Contribution of different causes of death to socioeconomic mortality inequality in Korean children aged 1–9: findings from a national mortality follow-up study. Journal of Epidemiology & Community Health. 2011;65(2):124-9.
86) Kaharuza F, Sabroe S, Scheutz F. Determinants of child mortality in rural Ugandan community. East African medical journal. 2001;78(12):630-6.
87) Kananura RM, Wamala R, Ekirapa-Kiracho E, Tetui M, Kiwanuka SN, Waiswa P, et al. A structural equation analysis on the relationship between maternal health services utilization and newborn health outcomes: a cross-sectional study in Eastern Uganda. BMC pregnancy and childbirth. 2017;17(1):98.
88) Katz J, West Jr KP, Khatry SK, Christian P, LeClerq SC, Pradhan EK, et al. Risk factors for early infant mortality in Sarlahi district, Nepal. Bulletin of the World Health Organization. 2003;81:717-25.
89) Kazembe L, Clarke A, Kandala N-B. Childhood mortality in sub-Saharan Africa: cross-sectional insight into small-scale geographical inequalities from Census data. BMJ open. 2012;2(5).
90) Keene Woods N, Reyes J, Chesser A. Infant mortality and race in Kansas: associations with women, infants, and children services. Journal of Primary Care & Community Health. 2016;7(3):194-8.
91) Kleinman JC, Pierre Jr MB, Madans JH, Land GH, Schramm WF. The effects of maternal smoking on fetal and infant mortality. American Journal of Epidemiology. 1988;127(2):274-82.
92) Ko Y-J, Shin S-H, Park S, Kim H-S, Lee J-Y, Kim K, et al. Effects of employment and education on preterm and full-term infant mortality in Korea. Public Health. 2014;128(3):254-61.
93) Kost K, Amin S. Reproductive and socioeconomic determinants of child survival: Confounded, interactive, and age-dependent effects. Social biology. 1992;39(1-2):139-50.
94) Koupil I, Rahu K, Rahu M, Karro H, Vågerö D. Major improvements, but persisting inequalities in infant survival in Estonia 1992–2002. The European Journal of Public Health. 2007;17(1):8-16.
95) Koupilova I, Bobak M, Holĉík J, Pikhart H, Leon DA. Increasing social variation in birth outcomes in the Czech Republic after 1989. American Journal of Public Health. 1998;88(9):1343-7.
96) Kuate DB. Causes and determinants of mortality under 2 years of age in subSaharan Africa: application of concurrent risk models. Cahiers quebecois de demographie. 1997;26(1):3.
97) Kusneniwar GN, Mishra AK, Balasubramanian K, Reddy PS. Determinants of Infant Mortality in a Developing Region in Rural Andhra Pradesh. Natl J Integr Res Med. 2013;4(4):20-26.
98) Lee H-Y, Do DV, Choi S, Trinh OTH, To KG. Trends and determinants of infant and under-five childhood mortality in Vietnam, 1986–2011. Global health action. 2016;9(1):29312.
99) Lemma P, Costa G, Demaria M, D'Ambrosio R, Magnani C. Social differences in infant mortality in a longitudinal Turin Study. Epidemiologia e Prevenzione. 1992;14(52):50-5.

100) Levandowski BA, Sharma P, Lane SD, Webster N, Nestor AM, Cibula DA, et al. Parental literacy and infant health: an evidence-based healthy start intervention. Health Promotion Practice. 2006;7(1):95-102.
101) Li C, Yan H, Zeng L, Dibley MJ, Wang D. Predictors for neonatal death in the rural areas of Shaanxi Province of Northwestern China: a cross-sectional study. BMC Public Health. 2015 Apr 16;15:387.
102) Lima Ede F, Sousa AI, Griep RH, Primo CC. Fatores de risco para mortalidade neonatal no município de Serra, Espírito Santo [Risk factors for neonatal mortality in the city of Serra, Espírito Santo]. Rev Bras Enferm. 2012 Jul-Aug;65(4):578-85. Portuguese.
103) Lugangira K, Kalokola F. Morbidity and mortality of children aged 2–59 months admitted in the Tanzania Lake Zone's public hospitals: a cross-sectional study. BMC research notes. 2017;10(1):502.
104) Luo Z-C, Wilkins R, Kramer MS. Effect of neighbourhood income and maternal education on birth outcomes: a population-based study. Cmaj. 2006;174(10):1415-20.
105) Machado CJ, Hill K. Maternal, neonatal and community factors influencing neonatal mortality in Brazil. Journal of biosocial science. 2005;37(2):193.
106) Machado, Carla Jorge, & Hill, Kenneth. (2003). Determinants of neonatal and post-neonatal mortality in the City of São Paulo. Revista Brasileira de Epidemiologia, 6(4), 345-358.
107) Macharelli CA, Oliveira LRd. Death risk profile of children under one year of age in an interior town of S. Paulo State, Brazil, in 1987. Revista de saude publica. 1991;25(2):121-8.
108) Majumder AK, May M, Pant PD. Infant and child mortality determinants in Bangladesh: Are they changing? Journal of Biosocial Science. 1997;29(4):385-99.
109) Malakar B, Roy SK. Effect of socio-economic characteristics on fertility and under-five mortality: examples from the Santals of Birbhum district, West Bengal, India. Anthropological Review. 2017;80(3):323-34.
110) Målqvist M, Hoa DPT, Persson L-Å, Ekholm Selling K. Effect of facilitation of local stakeholder groups on equity in neonatal survival; results from the NeoKIP trial in Northern Vietnam. PLoS One. 2015;10(12):e0145510.
111) Målqvist M, Nga NT, Eriksson L, Wallin L, Hoa DP, Persson LÅ. Ethnic inequity in neonatal survival: a case-referent study in northern Vietnam. Acta Paediatrica. 2011;100(3):340-6.
112) Målqvist M, Sohel N, Do TT, Eriksson L, Persson L-Å. Distance decay in delivery care utilisation associated with neonatal mortality. A case referent study in northern Vietnam. BMC Public Health. 2010;10(1):762.
113) Mavalankar DV, Trivedi C, Gray RH. Levels and risk factors for perinatal mortality in Ahmedabad, India. Bulletin of the World Health Organization. 1991;69(4):435.
114) Mbago MC. Some correlates of child mortality in the refugee populated regions in Tanzania. Journal of biosocial science. 1994;26(4):451-67.
115) Mengistu BA, Yismaw AE, Azene ZN, Mihret MS. Incidence and predictors of neonatal mortality among neonates admitted in Amhara regional state referral hospitals, Ethiopia: prospective follow up study. BMC Pediatr. 2020 Apr 1;20(1):142.
116) Miller JE, Trussell J, Pebley AR, Vaughan B. Birth spacing and child mortality in Bangladesh and the Philippines. Demography. 1992;29(2):305-18.

117) Mishra S, Ram B, Singh A, Yadav A. Birth order, stage of infancy and infant mortality in India. Journal of biosocial science. 2018;50(5):604-25.
118) Mohamoud YA, Kirby RS, Ehrenthal DB. Poverty, urban-rural classification and term infant mortality: a population-based multilevel analysis. BMC Pregnancy Childbirth. 2019 Jan 22;19(1):40.
119) Mombelli MA, Sass A, Molena CAF, Téston EF, Marcon SS. Risk factors for child mortality in towns of Paraná State (South Brazil), from 1997 to 2008. Rev Paul Pediatr. 2012;30(2):187-94.
120) Mondal MNI, Hossain MK, Ali MK. Factors influencing infant and child mortality: A case study of Rajshahi District, Bangladesh. Journal of Human Ecology. 2009;26(1):31-9.
121) Monteiro M. The effect of maternal education on the risk of infant mortality. Revista brasileira de estudos de populacao. 1990;7(1):74-86.
122) Morais Neto, Otaliba Libânio de, & Barros, Marilisa Berti de Azevedo. (2000). Fatores de risco para mortalidade neonatal e pós-neonatal na Região Centro-Oeste do Brasil: linkage entre bancos de dados de nascidos vivos e óbitos infantis. Cadernos de Saúde Pública, 16(2), 477-485.
123) Morrison J, Najman J, Williams G, Keeping J, Andersen M. Socio-economic status and pregnancy outcome. An Australian study. BJOG: An International Journal of Obstetrics & Gynaecology. 1989;96(3):298-307.
124) Nakhzari-Moghaddam M, Yavari P, Abadi A, Rostami-Gooran N. Association of socioeconomic risk factors with under 5-year mortality in Zabol, Iran, during 2011-2015. Med J Islam Repub Iran. 2019 Jun 26;33:61.
125) Naz S, Page A, Agho KE. Household air pollution and under-five mortality in India (1992–2006). Environmental Health. 2016;15(1):54.
126) Ng weshemi J, Urassa M, Isingo R, Mwaluko G, Ngalula J, Boerma T, et al. HIV impact on mother and child mortality in rural Tanzania. JAIDS-HAGERSTOWN MD-. 2003;33(3):393-404.
127) Obeidat N, Khader Y, Batieha A, Abdel Razeq N, Al-Sheyab N, Khassawneh M. Neonatal mortality in Jordan: secondary analysis of Jordan Population and Family Health Survey (JPFHS) data. The Journal of Maternal-Fetal & Neonatal Medicine. 2019;32(2):217-24.
128) Olsen O, Madsen M. Effects of maternal education on infant mortality and stillbirths in Denmark. Scandinavian journal of public health. 1999;27(2):128-36.
129) Palacio Chaverra A. The color of child survival in Colombia, 1955–2005. Ethnicity & health. 2018;23(2):207-20.
130) Pandey S, Lin Y. Adjusted effects of domestic violence, tobacco use, and indoor air pollution from use of solid fuel on child mortality. Maternal and child health journal. 2013;17(8):1499-507.
131) Parazzini F, Pirotta N, La Vecchia C, Bocciolone L, Fedele L. Determinants of perinatal and infant mortality in Italy. Revue d'épidémiologie et de santé publique. 1992;40(1):15.
132) Pardosi JF, Adair T, Rao C, Kosen S, Tarigan IU. Measuring subnational under-5 mortality: lessons from a survey in the Eastern Indonesian District of Ende. Asia Pacific Journal of Public Health. 2014;26(4):367-77.

133) Peña R, Liljestrand J, Zelaya E, Persson L-A. Fertility and infant mortality trends in Nicaragua 1964-1993. The role of women's education. Journal of Epidemiology & Community Health. 1999;53(3):132-7.
134) Peña R, Wall S, Persson L-A. The effect of poverty, social inequity, and maternal education on infant mortality in Nicaragua, 1988-1993. American journal of public health. 2000;90(1):64.
135) Pérez W, Eriksson L, Blandón EZ, Persson L-Å, Källestål C, Peña R. Comparing progress toward the millennium development goal for under-five mortality in León and Cuatro Santos, Nicaragua, 1990–2008. BMC pediatrics. 2014;14(1):9.
136) Pérez W, Peña R, Persson L-Å, Källestål C. Tracking progress towards equitable child survival in a Nicaraguan community: neonatal mortality challenges to meet the MDG 4. BMC public health. 2011;11(1):1-7.
137) Pickering H, Hayfs R, Ng'Andu N, Smith P. Social and environmental factors associated with the risk of child mortality in a peri-urban community in The Gambia. Transactions of the Royal Society of Tropical Medicine and Hygiene. 1986;80(2):311-6.
138) Price J, Willcox M, Kabudula CW, Herbst K, Kahn K, Harnden A. Home deaths of children under 5 years in rural South Africa: a population-based longitudinal study. Trop Med Int Health. 2019 Jul;24(7):862-878.
139) Qin C, Gould JB. The Asian birth outcome gap. Paediatric and perinatal epidemiology. 2006;20(4):279-89.
140) Rajna P, Mishra AK, Krishnamoorthy S. Impact of maternal education and health services on child mortality in Uttar Pradesh, India. Asia-Pacific Population Journal. 1998;13(2):27-38.
141) Ramos Padilla MA. [Infant mortality in Peru]. Salud Publica Mex. 1987;29(1):93-103.
142) Ranjan, M., L. Dwivedi, R. Mishra and Brajesh. "Infant mortality differentials among the tribal and non-tribal populations of Central and Eastern India." Journal of population studies 2 (2017).
143) Rao R, Chakladar B, Nair N, Kutty P, Acharya D, Bhat V, et al. Influence of parental literacy and socio-economic status on infant mortality. The Indian Journal of Pediatrics. 1996;63(6):795-800.
144) Rao SR, Pandey A, Shajy K. Child mortality in Goa: A cross-sectional analysis. Social Biology. 1997;44(1-2):101-10.
145) Ratnasiri AWG, Lakshminrusimha S, Dieckmann RA, Lee HC, Gould JB, Parry SS, Arief VN, DeLacy IH, DiLibero RJ, Basford KE. Maternal and infant predictors of infant mortality in California, 2007-2015. PLoS One. 2020 Aug 6;15(8):e0236877.
146) Regassa N. Infant mortality in the rural sidama zone, southern Ethiopia: Examining the contribution of key pregnancy and postnatal health care services. Journal of Nursing, Social Studies, Public Health and Rehabilitation. 2012;1(2):51-61.
147) Ribeiro, Adolfo Monteiro, Guimarães, Maria José, Lima, Marilia de Carvalho, Sarinho, Silvia Wanick, & Coutinho, Sonia Bechara. (2009). Risk factors for neonatal mortality among children with low birth weight. Revista de Saúde Pública, 43(2), 246-255. Epub February 13, 2009.
148) Roy NC, Kane TT, Barkat-e-Khuda. Socioeconomic and health implications of adult deaths in families of rural Bangladesh. Journal of Health, Population and Nutrition. 2001:291-300.

149) Ruelas-Orozco G, Guzman J, Malacara J. Perinatal mortality risk factors in a case-control study. Boletin medico del Hospital Infantil de Mexico. 1985;42(3):153.
150) Rychtaříková J, Demko GJ. Inequalities in infant survival: An analysis of Czech linked records. European journal of Population/Revue européenne de Démographie. 2001;17(4):323-42.
151) Rychtaríková J. Do maternal and paternal characteristics perform similar roles in adverse pregnancy outcome and infant survival? Acta Universitatis Carolinae: Geographica. 2001;36(1):77-94.
152) Sahoo M, Pradhan J. Using Three Delay Model to Understand the Social Factors Responsible for Neonatal Deaths Among Displaced Tribal Communities in India. J Immigr Minor Health. 2020.
153) Sahu D, Nair S, Singh L, Gulati B, Pandey A. Levels, trends & predictors of infant & child mortality among Scheduled Tribes in rural India. The Indian journal of medical research. 2015;141(5):709.
154) Sandiford P, Cassel J, Sanchez G, Coldham C. Does intelligence account for the link between maternal literacy and child survival? Social science & medicine. 1997;45(8):1231-9.
155) Santos HGd, Andrade SMd, Silva AMR, Carvalho WOd, Mesas AE. Risk factors for infant mortality in a municipality in southern Brazil: a comparison of two cohorts using hierarchical analysis. Cadernos de Saúde Pública. 2012;28(10):1915-26.
156) Sartorius B, Kahn K, Collinson MA, Vounatsou P, Tollman SM. Survived infancy but still vulnerable: spatial-temporal trends and risk factors for child mortality in rural South Africa (Agincourt), 1992-2007. Geospatial health. 2011;5(2):285.
157) Schellenberg JRA, Mrisho M, Manzi F, Shirima K, Mbuya C, Mushi AK, et al. Health and survival of young children in southern Tanzania. BMC public health. 2008;8(1):194.
158) Semba RD, de Pee S, Kraemer K, Sun K, Thorne-Lyman A, Moench-Pfanner R, et al. Purchase of drinking water is associated with increased child morbidity and mortality among urban slum-dwelling families in Indonesia. International Journal of Hygiene and Environmental Health. 2009;212(4):387-97.
159) Shamebo D, Sandström A, Muhe L, Freij L, Krantz I, Lönnberg G, et al. The Butajira project in Ethiopia: a nested case-referent study of under-five mortality and its public health determinants. Bulletin of the World Health Organization. 1993;71(3-4):389.
160) Shapiro GD, Bushnik T, Sheppard AJ, Kramer MS, Kaufman JS, Yang S. Paternal education and adverse birth outcomes in Canada. J Epidemiol Community Health. 2017;71(1):67-72.
161) Sharma RK. Causal pathways to infant mortality: linking social variables to infant mortality through intermediate variables. Journal of Health & Social Policy. 1998;9(3):15-28.
162) Shifa GT, Ahmed AA, Yalew AW. Socioeconomic and environmental determinants of under-five mortality in Gamo Gofa Zone, Southern Ethiopia: a matched case control study. BMC international health and human rights. 2018;18(1):14.
163) Shoham-Yakubovich I, BAREL V. Maternal education as a modifier of the association between low birthweight and infant mortality. International Journal of Epidemiology. 1988;17(2):370-7.
164) Silva ALd, Mathias TAdF. Independent risk factors associated with infant deaths. Acta Paulista de Enfermagem. 2014;27(1):48-55.

165) Singh A, Kumar A, Kumar A. Determinants of neonatal mortality in rural India, 2007-2008. PeerJ. 2013 May 28;1:e75.
166) Singh GK, Kposowa AJ. A comparative analysis of infant mortality in major Ohio cities: Significance of socio-biological factors. Applied behavioral science review. 1994;2(1):77-94.
167) Singh GK, Yu SM. Infant Mortality in the United States, 1915-2017: Large Social Inequalities have Persisted for Over a Century. Int J MCH AIDS. 2019;8(1):19-31.
168) Singh-Manoux A, Dugravot A, Smith GD, Subramanyam M, Subramanian S. Adult education and child mortality in India: the influence of caste, household wealth, and urbanization. Epidemiology. 2008;19(2):294.
169) Son M, An S-J, Kim Y-J. Trends of social Inequalities in the specific causes of Infant mortality in a nationwide birth cohort in Korea, 1995–2009. Journal of Korean medical science. 2017;32(9):1401-14.
170) Son M, Oh J, Choi YJ, Kong JO, Choi J, Jin E, et al. The Effects of the Parents' Social Class on Infant and Child Death among 1995-2004 Birth Cohort in Korea. Journal of Preventive Medicine and Public Health. 2006;39(6):469-76.
171) Spurlock C, Moser M, Flynn L. Regional differences in death rates among postneonatal infants in Kentucky, 1982-1985. The Journal of the Kentucky Medical Association. 1989;87(3):111-9.
172) Stoltenberg C, Magnus P, Lie RT, Daltveit AK, Irgens LM. Influence of consanguinity and maternal education on risk of stillbirth and infant death in Norway, 1967–1993. American Journal of Epidemiology. 1998;148(5):452-9.
173) Tawiah E. Child mortality differentials in Ghana: a preliminary report. Journal of biosocial science. 1989;21(3):349-55.
174) Teixeira GA, Costa FMdL, Mata MdS, Bittencourt Leite de Carvalho J, Souza NLd, Silva RARd. Risk factors for neonatal mortality in the life of first week. Revista de Pesquisa: Cuidado é Fundamental Online. 2016;8(1):4036-46.
175) Torres, V. E. R., Bertone, C. L., & Andrada, M. J. (2018). Brechas en la mortalidad infantil según nivel educativo de las madres en la Provincia de Córdoba. Estimación indirecta a partir de datos censales 2010. Revista De Salud Pública, 22(3), 37–47.
176) Torres, V. E. R., Bertone, C. L., & Andrada, M. J. (2018). Brechas en la mortalidad infantil según nivel educativo de las madres en la Provincia de Córdoba. Estimación indirecta a partir de datos censales 2010. Revista De Salud Pública, 22(3), 37–47.
177) Tzoumaka-Bakoula C, Lekea-Karanika V, Matsaniotis N, Golding J. The Greek national perinatal survey. II: Socioeconomic factors and perinatal mortality in Greece. Paediatric and perinatal epidemiology. 1989;3(1):41-52.
178) Tzoumaka-Bakoula C, Lekea-Karanika V, Matsaniotis N, Shenton T, Golding J. Are there gaps in the provision of perinatal care in Greece? Journal of Epidemiology & Community Health. 1989;43(4):319-23.
179) Victoria CG, Huttly SR, Barros FC, Lombardi C, Vaughan JP. Maternal education in relation to early and late child health outcomes: findings from a Brazilian cohort study. Social science & medicine. 1992;34(8):899-905.
180) Wahab A, Winkvist A, Stenlund H, Wilopo SA. Infant mortality among Indonesian boys and girls: effect of sibling status. Annals of tropical paediatrics. 2001;21(1):66-71.
181) Wang P, Lin R. Perinatal mortality in Taiwan. Public Health. 1999;113(1):27-33.

182) Wang X, Strobino DM, Guyer B. Differences in cause-specific infant mortality among Chinese, Japanese, and white Americans. American journal of epidemiology. 1992;135(12):1382-93.
183) Whitworth A, Stephenson R. Birth spacing, sibling rivalry and child mortality in India. Social science & medicine. 2002;55(12):2107-19.
184) Winbo I, Serenius F, Dahlquist G, Källén B. Maternal risk factors for cause-specific stillbirth and neonatal death. Acta obstetricia et gynecologica Scandinavica. 2001;80(3):235-44.
185) Yu Y, Liew Z, Wang A, Arah OA, Li J, Olsen J, Cnattingius S, Qin G, Obel C, Fu B, Li J. Mediating roles of preterm birth and restricted fetal growth in the relationship between maternal education and infant mortality: A Danish population-based cohort study. PLoS Med. 2019 Jun 14;16(6):e1002831.
186) Yudrika A, Zulkifli A, Muis M, editors. Risk Factors of Neonatal Mortality Incidence in Muna Regency in 2014. Proceedings of the 2nd International Conference on Medical and Health Informatics; 2018.

9. MR-BRT Methods for Parental Education Meta-Analysis.

This Appendix details the statistical model and fitting procedure used in the analysis. The article [4] has a more complete mathematical specification of the model.

(1) Mixed-Effects Model

We consider the following basic nonlinear mixed effects model:

$$\begin{aligned} \boldsymbol{y}_i &= \mathbf{X}_i\boldsymbol{\beta} + \mathbf{Z}_i\boldsymbol{u}_i + \boldsymbol{\epsilon}_i \\ \boldsymbol{u}_i &\sim N(\mathbf{0}, \boldsymbol{\Gamma}), \quad \boldsymbol{\Gamma} = \mathrm{diag}(\boldsymbol{\gamma}), \quad \boldsymbol{\epsilon}_i \sim N(\mathbf{0}, \boldsymbol{\Lambda}), \end{aligned} \tag{1}$$

where $\boldsymbol{y}_i \in \mathbb{R}^{n_i}$ is the vector of observations from the ith study, $\boldsymbol{\epsilon}_i \in \mathbb{R}^{n_i}$ are measurement errors with given covariance $\boldsymbol{\Lambda}$, $\boldsymbol{u}_i \in \mathbb{R}^{k_\gamma}$ are independent random effects, and $\mathbf{Z}_i \in \mathbb{R}^{n_i \times k_\gamma}$ is a linear map, and $\boldsymbol{\beta}$ are regression coefficients. The models F_i may be nonlinear.

To fit $(\boldsymbol{\beta}, \boldsymbol{\gamma})$ we solve the marginal likelihood problem

$$\min_{\boldsymbol{\beta},\boldsymbol{\gamma}} f(\boldsymbol{\beta}, \boldsymbol{\gamma}) := \sum_{i=1}^{m} \frac{1}{2}(\boldsymbol{y}_i - \mathbf{X}_i\boldsymbol{\beta})^\top(\mathbf{Z}_i\boldsymbol{\Gamma}\mathbf{Z}_i^\top + \boldsymbol{\Lambda}_i)^{-1}(\boldsymbol{y}_i - \mathbf{X}_i\boldsymbol{\beta}) + \frac{1}{2}\ln|\mathbf{Z}_i\boldsymbol{\Gamma}\mathbf{Z}_i^\top + \boldsymbol{\Lambda}_i|. \tag{2}$$

(2) Trimming outliers.

Least trimmed squares (LTS) is a robust estimator proposed by [2, 3] for the standard regression problem. Given the problem

$$\min_{\boldsymbol{\beta}} \sum_{i=1}^{n} \frac{1}{2}(y_i - \langle \mathbf{X}_i, \boldsymbol{\beta}\rangle)^2, \tag{3}$$

the LTS estimator minimizes the sum of *smallest* h residuals rather than all residuals. These estimators were initially introduced to develop linear regression estimators that have a high breakdown point (in this case 50%) and good statistical efficiency (in this case $n^{-1/2}$).[1] LTS estimators are robust against outliers, and arbitrarily large deviations that are trimmed do not affect the final $\hat{\boldsymbol{\beta}}$.

Rather than writing the objective in terms of order statistics, it is far simpler to extend the likelihood using an auxiliary variable $\mathbf{W}$:

$$\min_{\boldsymbol{\beta},\mathbf{W}} \sum_{i=1}^{n} w_i \left(\frac{1}{2}(y_i - \langle \mathbf{X}_i, \boldsymbol{\beta}\rangle)^2\right) \quad \text{s.t.} \quad \mathbf{1}^\top\mathbf{W} = h, \quad \mathbf{0} \le \mathbf{W} \le \mathbf{1}. \tag{4}$$

The set

$$\Delta_h := \left\{\mathbf{W} : \mathbf{1}^\top\mathbf{W} = h, \quad \mathbf{0} \le \mathbf{W} \le \mathbf{1}\right\} \tag{5}$$

is known as the *capped simplex*, since it is the intersection of the h-simplex with the unit box (see [1] for details). For a fixed $\boldsymbol{\beta}$, the optimal solution of (4) with respect to $\mathbf{W}$ assigns weight 1 to each of the smallest h residuals, and 0 to the rest. Problem (4) is solved *jointly* in $(\boldsymbol{\beta}, \mathbf{W})$, simultaneously finding the regression estimate and classifying the observations into inliers and outliers. This joint strategy

[1] Breakdown refers to the percentage of outlying points which can be added to a dataset before the resulting M-estimator can change in an unbounded way. Here, outliers can affect both the outcomes and training data (features).

makes LTS different from post-hoc analysis, where a model a fit first with all data, and then outliers are detected using that estimate.

To explain how trimming enters the marginal likelihood problem, we focus on a single group term from the ML likelihood (2):

$$\left(\frac{1}{2}(\boldsymbol{y}_i - \mathbf{X}_i\boldsymbol{\beta})^\top(\mathbf{Z}_i\boldsymbol{\Gamma}^{-1}\mathbf{Z}_i^\top + \boldsymbol{\Lambda}_i)^{-1}(\boldsymbol{y}_i - \mathbf{X}_i\boldsymbol{\beta}) + \frac{1}{2}\ln|\mathbf{Z}_i\boldsymbol{\Gamma}^{-1}\mathbf{Z}_i^\top + \boldsymbol{\Lambda}_i|\right).$$

We introduce auxiliary variables $\mathbf{W}_i \in \mathbb{R}^{n_i}$, and define

$$\boldsymbol{r}_i := \boldsymbol{y}_i - \mathbf{X}_i\boldsymbol{\beta}, \quad \mathbf{W}_i := \mathrm{diag}(\mathbf{W}_i), \quad \sqrt{\mathbf{W}_i} := \mathrm{diag}(\sqrt{\mathbf{W}_i}).$$

We now form the objective

$$\frac{1}{2}\boldsymbol{r}_i^\top\sqrt{\mathbf{W}_i}\left(\sqrt{\mathbf{W}_i}\mathbf{Z}_i\boldsymbol{\Gamma}^{-1}\mathbf{Z}_i^\top\sqrt{\mathbf{W}_i} + \boldsymbol{\Lambda}_i^{\odot\mathbf{W}_i}\right)^{-1}\sqrt{\mathbf{W}_i}\boldsymbol{r}_i + \frac{1}{2}\ln\left|\sqrt{\mathbf{W}_i}\mathbf{Z}_i\boldsymbol{\Gamma}^{-1}\mathbf{Z}_i^\top\sqrt{\mathbf{W}_i} + \boldsymbol{\Lambda}_i^{\odot\mathbf{W}_i}\right|, \quad (6)$$

where $^\odot$ denotes the elementwise power operation:

$$\boldsymbol{\Lambda}_i^{\odot\mathbf{W}_i} := \begin{bmatrix} (\lambda_{1j})^{w_{i1}} & 0 & \dots & 0 \\ 0 & \ddots & \ddots & \vdots \\ 0 & \dots & 0 & (\lambda_{in_i})^{w_{in_i}}. \end{bmatrix} \quad (7)$$

When $w_{ij} = 1$, we recover the contribution of the ijth observation to the original likelihood. As $w_{ij} \downarrow 0$, The ijth contribution to the residual is correctly eliminated by $\sqrt{w_{ij}} \downarrow 0$. The jth row and column of $\sqrt{\mathbf{W}_i}\mathbf{Z}_i\boldsymbol{\Gamma}^{-1}\mathbf{Z}_i^\top\sqrt{\mathbf{W}_i}$ both go to 0, while the jth entry of $\boldsymbol{\Lambda}_i^{\odot\mathbf{W}_i}$ goes to 1, which effectively removes all impact of the jth point on the covariance matrix.

(3) Posterior Variance-Covariance Matrix

In the meta-regression setting, the model we consider is given by:

$$\boldsymbol{y} = \boldsymbol{X}\boldsymbol{\theta} + \boldsymbol{U}\boldsymbol{\gamma} + \boldsymbol{\epsilon}, \quad \boldsymbol{\epsilon} \sim N(0, \sigma^2\boldsymbol{I}),$$

and the estimate for $\boldsymbol{\theta}$ at the true value forγ is given by

$$\hat{\boldsymbol{\theta}} = (\boldsymbol{X}^T\boldsymbol{V}(\gamma)^{-1}\boldsymbol{X})^{-1}\boldsymbol{X}^T\boldsymbol{V}(\gamma)^{-1}\boldsymbol{y},$$

with its variance is computed by

$$\boldsymbol{V}(\hat{\boldsymbol{\theta}}) == (\boldsymbol{X}^T\boldsymbol{V}(\gamma)^{-1}\boldsymbol{X})^{-1}. \quad (8)$$

To obtain an estimate of this matrix we can replace $\boldsymbol{\gamma}$ by its estimate $\hat{\boldsymbol{\gamma}}$.

Sampling from this distribution, a single measurement $\hat{y}_i$ for given values of the design matrix $\boldsymbol{x}$ has variance

$$V(y_i) = \boldsymbol{x}^T(\boldsymbol{X}^T\boldsymbol{V}(\gamma)^{-1}\boldsymbol{X})^{-1}\boldsymbol{x}.$$

References

[1] A. Aravkin and D. Davis. Trimmed statistical estimation via variance reduction. *Mathematics of Operations Research*, 2019.

[2] P. J. Rousseeuw. Multivariate Estimation with High Breakdown Point. *Mathematical statistics and applications*, 8:283–297, 1985.

[3] P. J. Rousseeuw and C. Croux. Alternatives to the median absolute deviation. *Journal of the American Statistical association*, 88(424):1273–1283, 1993.

[4] P. Zheng, R. Barber, R. J. Sorensen, C. J. Murray, and A. Y. Aravkin. Trimmed constrained mixed effects models: Formulations and algorithms. *Journal of Computational and Graphical Statistics*, 00(0): 1–13, 2021. doi.org/10.1080/10618600.2020.1868303
